# Inorganic-Ion-Stabilized Au Nanoparticles Supported on SiO_2_: Synthesis, Physicochemical Characterization, and Proof-of-Concept Raman Response Toward Methylene Blue

**DOI:** 10.3390/nano16191243

**Published:** 2026-10-01

**Authors:** Jorge Luis Mendoza Tellez, Omar E. Uribe-Juárez, Bruno Landeros-Rivera, Alba Adriana Vallejo-Cardona, Diana M. Osorio-Londoño, Federico González García, Francisco Tzompantzi, Raúl Pérez-Hernández, Rebeca Sosa Fonseca, Luis Alberto Moreno Ruiz, Ana María Soto-Estada, Iris N. Serratos

**Affiliations:** 1 Departamento de Química, Universidad Autónoma Metropolitana-Iztapalapa, Av. San Rafael Atlixco 186, Col. Vicentina, México City 09340, Mexico; cbi2173048089@izt.uam.mx (J.L.M.T.); fjtz@xanum.uam.mx (F.T.); 2 Departamento de Física, Universidad Autónoma Metropolitana-Iztapalapa, Av. San Rafael Atlixco 186, Col. Vicentina, México City 09340, Mexico; ouribe@xanum.uam.mx (O.E.U.-J.); rebe@xanum.uam.mx (R.S.F.); 3 Departamento de Química Inorgánica, Facultad de Química, Universidad Nacional Autónoma de México, Ciudad Universitaria, Coyoacán, México City 04510, Mexico; brunolanderos@quimica.unam.mx; 4 Biotecnología Médica y Farmacéutica, Centro de Investigación y Asistencia en Tecnología y Diseño del Estado de Jalisco, Av. Normalistas 800, Colinas de La Normal, Guadalajara 44270, Mexico; avallejo@ciatej.mx; 5 Departamento de Ingeniería Eléctrica, Universidad Autónoma Metropolitana-Iztapalapa, Av. San Rafael Atlixco 186, Col. Vicentina, México City 09340, Mexico; dmol@xanum.uam.mx; 6 División de Ingeniería Mecánica e Industrial, Facultad de Ingeniería, Universidad Nacional Autónoma de México, Ciudad Universitaria, Coyoacán, México City 04510, Mexico; 7 Departamento de Ingeniería de Procesos e Hidráulica, Universidad Autónoma Metropolitana, México City 04510, Mexico; fgonzalez@izt.uam.mx; 8 Instituto Nacional de Investigaciones Nucleares, Car. Méx.-Tol. s/n, La Marquesa, Ocoyoacac 52750, Mexico; raul.perez@inin.gob.mx; 9 Centro de Nanociencias y Micro y Nanotecnologías (CNMN) del Instituto Politécnico Nacional, Av. Luis Enrique Erro s/n, Nueva Industrial Vallejo, Gustavo A. Madero, México City 07700, Mexico; lmorenor@ipn.mx

**Keywords:** Stöber method, silicon oxide (SiO_2_), gold nanoparticles (AuNPs), inorganic stabilization, surface modification, surface-enhanced Raman scattering (SERS), methylene blue (MB)

## Abstract

Silica nanostructures are attractive platforms for hybrid nanomaterials because of their chemical stability and versatile surface functionalization. Here, spherical SiO_2_ nanoparticles were synthesized by a modified Stöber method, functionalized with (3-aminopropyl)triethoxysilane (APTES), and decorated with gold nanoparticles (AuNPs) formed using an inorganic stabilization strategy involving OH^−^ and Cl^−^ ions, without conventional organic AuNP capping agents. Complementary characterization by Fourier-transform infrared spectroscopy (FTIR), ultraviolet–visible diffuse reflectance spectroscopy (UV–VIS DRS), X-ray diffraction (XRD), scanning electron microscopy (SEM), and transmission electron microscopy (TEM) supported the sequential surface modification and formation of the SiO_2_–APTES–AuNPs hybrid material. TEM yielded average diameters of 100.81 ± 1.74 nm for SiO_2_ nanoparticles (*n* = 50) and 2.00 ± 0.31 nm for supported AuNPs (*n* = 80). Methylene blue (MB) was subsequently used for a proof-of-concept Raman evaluation. At 10^−3^ and 10^−4^ M MB, relative Raman signal-amplification ratios of approximately 7.5 and 3.5, respectively, were obtained from the integrated response near 1620 cm^−1^; these values are not conventional surface-enhanced Raman scattering (SERS) enhancement factors. At 10^−6^ M, a fluorescence-like background was observed without establishing metal-enhanced fluorescence. Density functional theory calculations using an Au_32_ cluster provided qualitative insight into local S···Au and π···Au interactions. Overall, these results establish an inorganic SiO_2_-AuNPs as a physicochemically characterized hybrid nanomaterial prepared through an inorganic AuNP-stabilization strategy and support its further investigation in Raman-based applications.

## 1. Introduction

In the field of nanoscience, nanostructured silica has gained significance because of its physicochemical properties, including mechanical strength, chemical stability, biocompatibility, and synthetic versatility [1]. This nanomaterial, composed of chains of silicon oxide (SiO_2_), is characterized by the presence of pores ranging from mesopores to micropores in diameter. This distinctiveness provides two distinct domains: an external surface and an interior within the pores [2].

**Figure 1:**
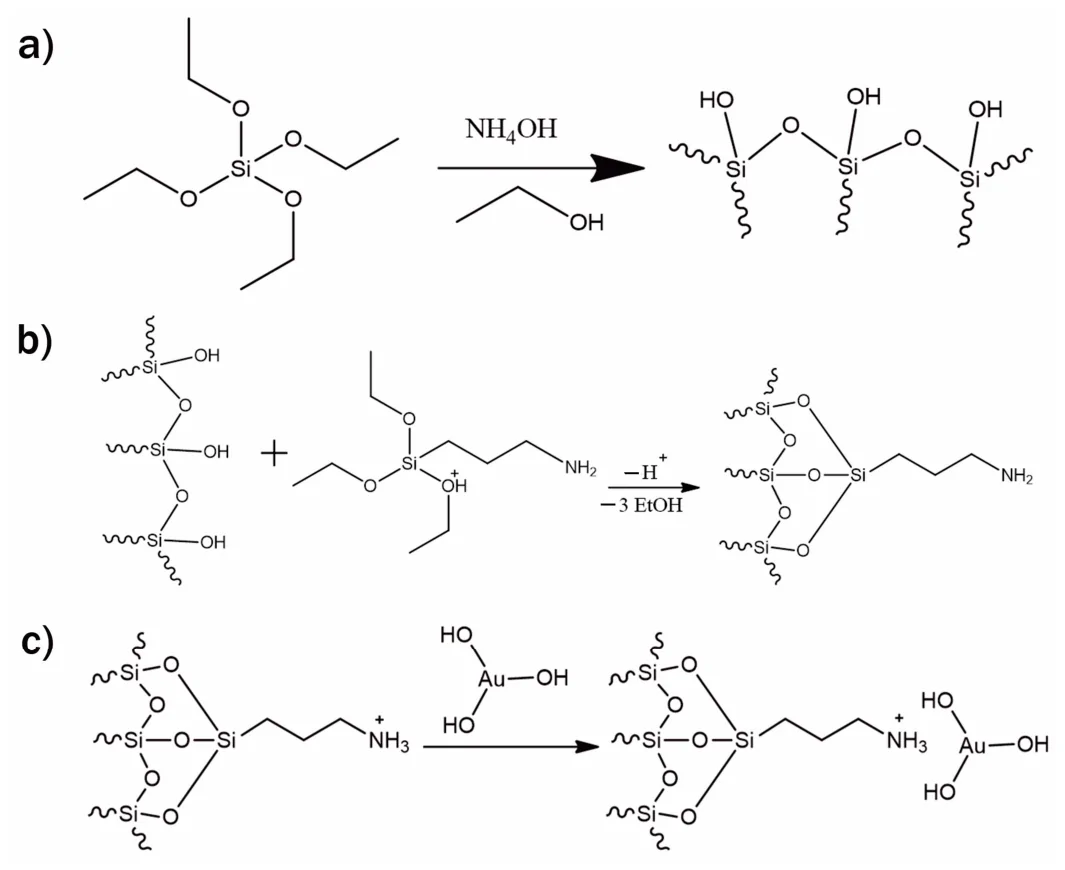
Schematic representation of the overall SiO_2_–APTES–AuNPs preparation route: (**a**) SiO_2_ formation by the modified Stöber process; (**b**) APTES surface functionalization; and (**c**) deposition of the gold precursor followed by AuNP formation using the inorganic stabilization strategy.

The Stöber process is among the most commonly used sol–gel methods for preparing monodisperse SiO_2_ nanoparticles [3]. This method uses a tetraethoxysilane (TEOS) precursor that undergoes hydrolysis in a basic medium composed of ethanol, ammonium hydroxide, and water (Figure 1a) [4]. Many research groups have applied these monodisperse silica colloids as model materials in various applications [5]. Sacks and Tseng utilized these colloids to pack membranes with ordered structures and investigated their sintering behavior [6]. Unger et al. employed submicrometer silica colloids as filler materials for capillary chromatography [7].

The compound APTES is linked to a silicon atom substituted by three hydroxyl groups. This compound is typically used in surface functionalization processes through substitution reactions on metal oxides such as SiO_2_ and titanium oxide (TiO_2_) [8]. The SiO_2_ nanoparticles possess surface hydroxyl groups, which act as nucleophiles to undergo substitution reactions, assisted by the acidic catalysis of APTES. In this manner, the silicon from APTES binds to the nanoparticle surface, liberating ethanol into the medium (Figure 1b) [9]. In this study, the surface functionalization of SiO_2_ nanoparticles was performed through alcoholic substitution assisted by acidic catalysis of acetic acid.

Gold nanoparticles (AuNPs) exhibit diverse physical, chemical, and biological properties depending on their shape and size. Therefore, their synthesis has been extensively explored to systematize the conditions for obtaining nanoparticles with various characteristics [10]. AuNPs can be easily functionalized with multiple ligands, such as antibodies, polymers, drugs, and genetic materials, etc. [11], making them particularly valuable for applications in the biomedical and food sectors. AuNPs are typically produced in aqueous media by reducing tetrachloroauric acid (HAuCl_4_) to form clusters. These are then stabilized during the growth stage by interactions with organic molecules on the nanoparticle surface [12].

In this study, SiO_2_ nanoparticles were coated with AuNPs. For this purpose, auric hydroxide must be prepared through a primary hydrolysis reaction [13]. Metallic hydroxides tend to have negative charge density and can be utilized to couple gold compounds through electrostatic interactions with species that exhibit partial or formal positive charges [14].

One of the functional groups commonly used for the coupling of gold precursors is primary amines, as they have a pKa between 9 and 10. Consequently, in an aqueous medium with pH values below the pKa, they are acidic, representing a positively charged species [15]. SiO_2_ nanoparticles are previously coated with amino groups to facilitate coating through electrostatic interactions between the hydroxide groups of the complex and the surface amino groups of the nanoparticles (Figure 1c) [14].

Once the gold compound adheres to the SiO_2_ nanoparticles, reducing species are added to promote the formation of gold nanoparticles [14]. The gold nanoparticles cluster and grow on the surface of the support material.

Beyond their structural and chemical versatility, SiO_2_ nanostructures coated with plasmonic metals have emerged as attractive candidates for surface-enhanced Raman scattering (SERS) [1,10]. In these hybrid materials, the silica framework provides mechanical and morphological stability and a functionalizable support for nanoparticle deposition, whereas gold domains provide plasmonically active surface sites through localized surface plasmon resonance (LSPR) [1,10,13,14]. The magnitude of plasmonic coupling depends strongly on AuNP size, spacing, aggregation state, and local architecture; these parameters were not systematically optimized in the present study. The use of an inorganic stabilization strategy during AuNP formation is relevant because conventional organic AuNP capping agents, such as citrate or polyvinylpyrrolidone, may affect analyte access to the metal surface or contribute spectral background [16]. Size-dependent plasmonic behavior of gold nanostructures has also been discussed theoretically [17].

Methylene blue (MB) is frequently used as a molecular probe in Raman and SERS studies because of its well-resolved vibrational spectrum and strong absorption in the visible region [17,18,19,20]. In the present study, MB was used for a proof-of-concept evaluation of the spectroscopic response of the synthesized SiO_2_–APTES–AuNPs material. In metal-assisted spectroscopies, the measured response depends on analyte concentration, adsorption geometry, fluorophore–metal distance, and the local plasmonic environment [17,21]. Interparticle spacing between AuNPs can strongly influence electromagnetic coupling and the formation of local high-field regions; however, the present study did not experimentally optimize or quantitatively determine such coupling. Accordingly, the concentration-dependent Raman spectra are interpreted conservatively, taking into account possible conventional Raman contributions from bulk or unadsorbed MB and the non-uniform analyte distribution produced by drop-casting. The elevated background observed at low MB concentration is described as fluorescence-like and is not considered direct evidence of metal-enhanced fluorescence (MEF).

A molecular-level interpretation of the spectroscopic behavior also requires consideration of interactions between MB and the gold surface. Density functional theory (DFT) can provide qualitative information on preferred adsorption motifs and local molecular–metal contacts [22,23]. In the present work, the Au_32_ cluster is used only as a simplified local-interaction model. Because it is substantially smaller than the experimentally observed ~2 nm AuNPs and does not include the SiO_2_–APTES support, it cannot reproduce the size-dependent electronic/plasmonic properties, interparticle coupling, or complete architecture of the experimental material. The calculations are therefore used to identify plausible MB–Au contacts that may influence molecular orientation and vibrational response, rather than to establish quantitative correspondence with the experimental nanostructure.

In this work, the objective was to synthesize and physicochemically characterize SiO_2_-supported AuNPs prepared using an inorganic stabilization strategy for the gold nanoparticles, without conventional organic AuNP capping agents. Raman measurements with MB were included as a proof-of-concept spectroscopic evaluation of the resulting material. DFT, quantum theory of atoms in molecules (QTAIM), and noncovalent interaction (NCI) analyses were used to examine local MB-Au interactions. The study does not claim comprehensive analytical SERS validation or experimentally demonstrated metal-enhanced fluorescence (MEF).

## 2. Materials and Methods

### 2.1. Reagents and Equipment

Anhydrous ethyl alcohol (99.5%, Sigma Aldrich), deionized water, ammonium hydroxide (28.0–30.0%, Sigma Aldrich), Tetraethyl orthosilicate (98.0%, Sigma Aldrich), chloroauric acid (99.0%, Sigma Aldrich), (3-aminopropyl)triethoxysilane (99.0%, Sigma Aldrich). Perkin Elmer FT-IR Spectrum GX (PerkinElmer, Inc., Waltham, MA, USA), UV-VIS Lambda 40 (PerkinElmer, Inc., Waltham, MA, USA), Jeol-SEM 7600F (JEOL Ltd., Tokyo, Japan).

### 2.2. Synthesis of SiO_2_ Spheres

We prepared SiO_2_ nanoparticles by modifying the Stöber method. We prepared a solution containing 2.2 M water (0.04 L), 11.7 M ethanol (0.68 L), 0.53 M (0.036 L) ammonium hydroxide and 0.30 M TEOS (0.068 L). The solution was kept under constant stirring (400 rpm) at 40 °C for 24 h to carry out the hydrolysis and condensation processes of TEOS.

The nanoparticles were meticulously washed with deionized water. The colloidal solution was subjected to precise centrifugation at 4000 rpm for 10 min, and this procedure was meticulously repeated three times using deionized water. A final wash was carried out using ethanol. The SiO_2_ nanoparticles were dried at 110 °C for 2 h and pulverized to obtain a fine powder. The obtained product was then characterized using Fourier-transform infrared spectroscopy (FTIR), ultraviolet–visible diffuse reflectance spectroscopy (UV–VIS DRS), X-ray diffraction (XRD), scanning electron microscopy (SEM), and transmission electron microscopy (TEM).

### 2.3. Functionalization

We functionalized SiO_2_ nanoparticles with -NH_2_ groups following the methodology of Netzahual Lopantzi et al. (2019), with the reaction conditions modified to achieve the desired coating [14].

Five SiO_2_ functionalization trials were evaluated during optimization. Under the selected conditions, the SiO_2_ nanoparticles (0.5 g) were suspended in an ethanol–water medium (1:1 *v*/*v*) at a 10 mg/mL ratio to a final volume of 50 mL. Afterward, 0.05% acetic acid (25 µL) and 5% APTES (2.5 mL) relative to the total volume of the colloid were added. Under these conditions, an excess of APTES molecules was ensured for the nanoparticle surface coating. To achieve homogeneity, the mixture was sonicated for 40 min and then reacted at 90 °C for 1 h under constant stirring of at least 400 rpm.

The functionalized SiO_2_ nanoparticles were subsequently washed with ethanol. We performed three cycles of centrifugation at 4000 rpm for 10 min. The precipitate was then recovered and suspended in ethanol at a concentration of 0.3 g/mL. Finally, a portion of the suspension was dried at 90 °C for two hours. We characterized the SiO_2_-APTES nanoparticles by FTIR, SEM, and TEM to verify the expected changes in composition, morphology, and size of the SiO_2_-APTES nanoparticles.

### 2.4. Gold Coating

For the gold coating of SiO_2_-APTES nanoparticles, we prepared a 6.35 mM HAuCl_4_ solution (10 mL) and placed it in an ice bath under constant stirring. Upon reaching 5–10 °C, 4.0 mL of 0.1 M NaOH (0.4 mmol) was added dropwise, and the mixture was stirred until a faint yellow solution was obtained. Once auric hydroxide (Au(OH)_3_) was formed, 0.5 mL of the SiO_2_-APTES suspension (0.3 g/mL) was added. The system was heated in a water bath at 70 °C for 30 min to promote the formation and interaction between the AuNPs and the SiO_2_–APTES nanoparticle surface. The SiO_2_–APTES–AuNP particles were then washed with deionized water by three centrifugation cycles at 800 rpm for 20 min, the precipitate was recovered, and it was resuspended in 10 mL of water. Afterwards, 224.4 µL of 0.5 M NH_4_OH (approximately 0.11 mmol) was added as the inorganic stabilizing solution. The gold-coated nanoparticles were dried at room temperature under nitrogen gas (N_2_). The three water-washing/centrifugation cycles constitute the purification procedure used in this study; they lessen the unbound species.

### 2.5. Characterization

After the synthesis of the SiO_2_–APTES–AuNPs, their surface chemistry was evaluated by FTIR using a PerkinElmer FT-IR Spectrum GX spectrometer (PerkinElmer, Inc., Waltham, MA, USA). The spectra were collected between 4000 and 400 cm^−1^. The appearance of the characteristic bands associated with APTES confirmed the successful functionalization of the nanoparticle surface.

The SiO_2_–APTES–AuNPs were characterized by UV–VIS spectroscopy using a Lambda 40 spectrophotometer (PerkinElmer, Inc., Waltham, MA, USA) in reflection mode with a solid-sample accessory. Measurements were carried out from 200 to 1200 nm. The plasmonic absorption observed near 523 nm is interpreted together with the complementary TEM and XRD results as evidence supporting AuNPs formation; the UV–VIS result is not treated as an isolated proof of the gold nanostructure.

Before the XRD patterns were acquired, we dried the samples by applying five thermal cycles of 3 min at 100 °C to remove residual moisture. Once the powders were dried, we recorded the XRD patterns using a Bruker D8 diffractometer configured to scan them from 5° to 90° in 2θ. The measurements were performed with a Cu Kα radiation source (λ = 1.54 Å), and the diffraction data were collected in 8407 steps with a counting time of 0.2 s per step.

Scanning and transmission electron microscopy were used to examine the morphology of the samples in each part of the methodology. SEM images of the nanoparticles were acquired using a JEOL JSM-7600F field-emission scanning electron microscope operating at 15 kV. Additionally, TEM analysis was performed with a JEOL transmission electron microscope operated at an accelerating voltage of 200 kV. The TEM micrographs reported in this study were acquired in bright-field imaging mode.

The samples for Raman measurement were prepared using the drop method. We made each sample by dropping 10 µL of SiO_2_–APTES–AuNPs onto the sample holder and drying it with five thermocycles of 1 min at 100 °C. After 10 µL of blue methylene solution was added to the surface of the SiO_2_–APTES–AuNPs, the final samples were dried using the same thermal-treatment. The samples were prepared with different concentrations MB from 1 × 10^−3^ M to 1 × 10^−6^ M.

The Raman response of the substrate was evaluated using MB at three different concentrations (10^−3^, 10^−4^, and 10^−6^ M). This concentration range was selected based on the concentration-dependent aggregation behavior of MB in aqueous media, where monomers progressively form dimers and higher-order aggregates as the concentration increases, as well as on the reported lack of fluorescence from MB aggregates, at least within the 625–850 nm spectral range [18,19].

Considering that the analysis of the absorption spectrum performed by Pominova et al. (2023) [19] indicated weak aggregation at concentrations above 0.01 mM (10^−5^ M), 10^−3^ M and 10^−4^ M were selected as concentrations above this reported aggregation threshold, whereas 10^−6^ M was selected as a concentration below it.

Raman spectra were acquired using a WITec Alpha 300 RA+ confocal micro-Raman spectrometer (Oxford Instruments, Ulm, Germany) with a 532 nm laser, with an incident power of 15 µW at the sample surface. These measurements were performed using a Zeiss EC Epiplan 50× objective (Carl Zeiss, Oberkochen, Germany) (NA 0.75), with an integration time of 4 s and 10 accumulations per spectrum.

Baseline correction was performed using the adaptive iteratively reweighted penalized least squares (airPLS) algorithm for the spectra acquired at 10^−3^ and 10^−4^ M. No baseline correction was applied to the spectra acquired at 10^−6^ M in order to preserve the fluorescence-like background observed at this concentration. No additional spectral processing was applied. Spectral data were handled using RamanSPy (version 0.2.10) [24]. Signal amplification was assessed using relative intensity ratios based on the integrated area of the Raman band at approximately 1620 cm^−1^ [17]. The area under the curve was calculated using the trapezoidal rule over the 1580–1650 cm^−1^ spectral range using NumPy (version: 1.26.4).

### 2.6. Quantum Mechanical Calculations

Geometry optimizations of the gold nanoparticle-methylene blue complex (AuNP-MB) were performed with the r2-SCAN-3c composite model [25]. The resulting geometries were reoptimized with the PBE0-D3(BJ)/def2-TZVP level of theory. Both geometry optimization processes were performed with Orca 5.0.3 [26]. The plane of the MB molecule was placed parallel or perpendicular to one of the faces of the AuNP, which was modeled using the Au_32_ icosahedral structure reported in the literature [27]. In parallel configurations, either the N or the S atoms were placed directly with one of the gold atoms of the AuNPs. Given that r2-SCAN-3c provided accurate geometries that differ slightly from those obtained from PBE0, a vibrational analysis and calculation of Raman activities of MB and AuNP-MB was performed with this method [25]. To perform a direct comparison of the MB and AuNP-MB spectra, the calculated spectra for both species were normalized. The topology of the electron density of the optimized structures was studied by means of the quantum theory of atoms in molecules (QTAIM) framework [28] using the AIMALL program (AIMAll (Version 19.10.12), Todd A. Keith, TK Gristmill Software, Overland Park, KS, USA, 2019 (aim.tkgristmill.com), and the electron localization function [29] which was analyzed with Multiwfn version 3.8 [30]. Additionally, the noncovalent interaction index (NCI) [31] of the optimized structures was analyzed with NCIPLOT4 version 4.2.1 alpha [32] using the promolecular approach. The Au_32_ cluster is a simplified local-interaction model and is smaller than the experimental AuNPs observed by TEM. It does not include the SiO2-APTES support and is therefore not used to reproduce size-dependent plasmonic properties or the complete experimental nanostructure.

## 3. Results and Discussion

### 3.1. FTIR Analysis

#### 3.1.1. SiO_2_ Nanoparticles

**Figure 2:**
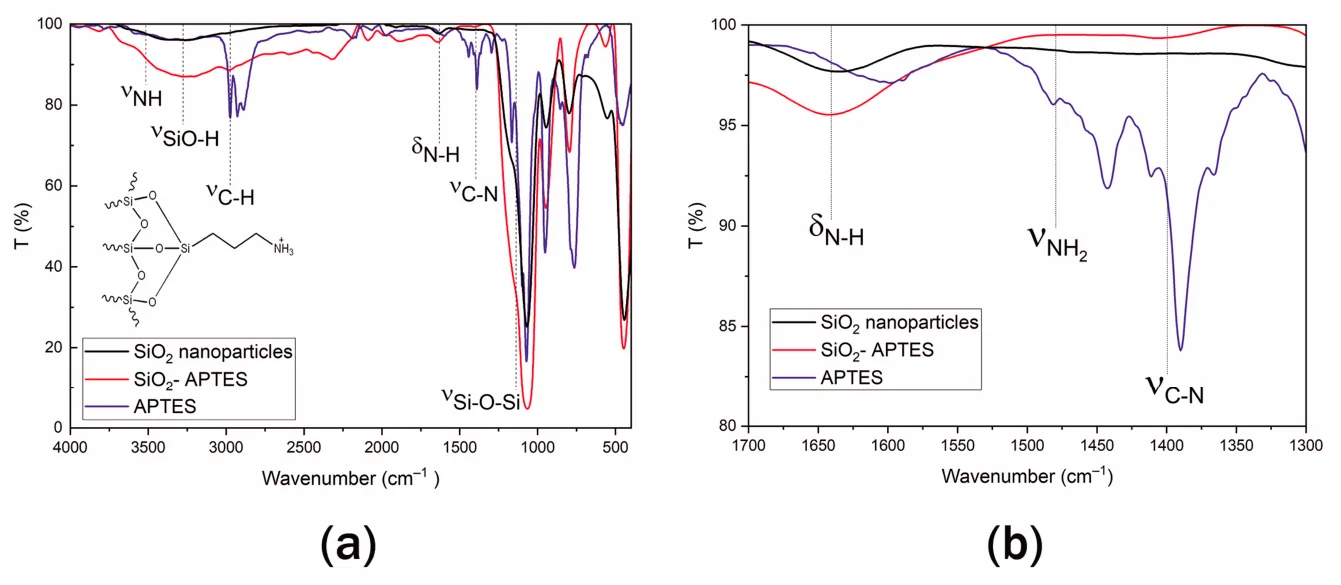
FTIR spectra of SiO_2_ nanoparticles, SiO_2_–APTES nanoparticles, and APTES. (**a**) FTIR spectra recorded over the range of 4000–400 cm^−1^ for SiO_2_ nanoparticles (black), SiO_2_–APTES nanoparticles (red), and APTES (blue). (**b**) Enlarged view of the 1700–1300 cm^−1^ region, highlighting the spectral features associated with APTES functionalization. The characteristic vibrational bands are indicated in the spectra.

FTIR analysis of the SiO_2_ nanoparticles was performed using a Perkin Elmer FT-IR Spectrum GX, and the equipment was set to wavenumbers from 400 to 4000 cm^−1^. The FTIR spectrum (Figure 2a) reveals a broad band at 3250 cm^−1^, assignable to a stretching vibration, corresponding to surface Si-O-H group bonds of the nanoparticle, pre-sent in the spherical morphology of SiO_2_. Additionally, the presence of physisorption water in the sample was evidenced by the band located at 1629 cm^−1^ [33], corresponding to bending vibrations of H-O-H bonds.

Furthermore, a highly intense band located at 1071 cm^−1^ denotes asymmetric stretching of Si–O–Si bonds. Moreover, a band at 952 cm^−1^ is attributed to the stretching vibrations of Si–OH bonds. The spectrum also shows a band at 798 cm^−1^, which is assignable to symmetric stretching vibrations of Si–O–Si bonds. Finally, a highly intense band at 450 cm^−1^ corresponds to the bending vibrations of O–Si–O bonds [34].

All the prepared SiO_2_ samples exhibited similar characteristic bands associated with Si–O vibrations in their FTIR spectra (Supplementary Figure S1).

#### 3.1.2. SiO_2_–APTES

The FTIR spectrum obtained from the functionalized nanoparticles was overlaid with the FTIR spectrum of the APTES reagent (Figure 2) to investigate changes in the nanoparticles directly associated with the APTES coating during synthesis.

In the FTIR spectrum of the functionalized nanoparticles, we identified specific bands that provide detailed information about the changes in the SiO_2_ nanoparticles. The OH bond vibrations cover the stretching bands of N–H bonds. However, there are perceptible bands of NH stretching in the 3200–3400 cm^−1^ region; these include bands at 3269 cm^−1^ and 3340 cm^−1^, which are considered characteristic of the stretching vibrations of N–H bonds in primary amines, and a peak at 2983 cm^−1^, which is at-tributed to the stretching vibrations of C–H bonds [35]. Moreover, we obtained a band at 1640 cm^−1^ related to the bending vibrations of N–H bonds and a weak band at 1408 cm^−1^ corresponding to the stretching vibrations of C–N bonds (Figure 2b). The attenuation of the aliphatic C–H stretching bands between 2800 and 3000 cm^−1^ in SiO_2_-APTES relative to neat APTES can be attributed to hydrolysis and condensation of the ethoxy groups during surface functionalization, resulting in Si–O–Si bond formation and ethanol elimination [36]. Consequently, the contribution of aliphatic C–H vibrations decreases in the grafted APTES layer. The band near 1640 cm^−1^ is associated with NH_2_ bending vibrations and may be influenced by hydrogen bonding between NH_2_ and surface silanol groups. This region also overlaps with the H-O-H bending vibration of physisorbed water observed near 1629 cm^−1^ in pristine SiO_2_.

Overall, the presence of bands associated with primary NH_2_ groups and aliphatic CH_2_ vibrations, together with the spectral changes relative to neat APTES, supports the successful functionalization of the SiO_2_ nanoparticle surface with APTES. The bands corresponding to amine groups were consistently observed after evaluating different functionalization conditions (Supplementary Figure S2).

### 3.2. UV–VIS Analysis

**Figure 3:**
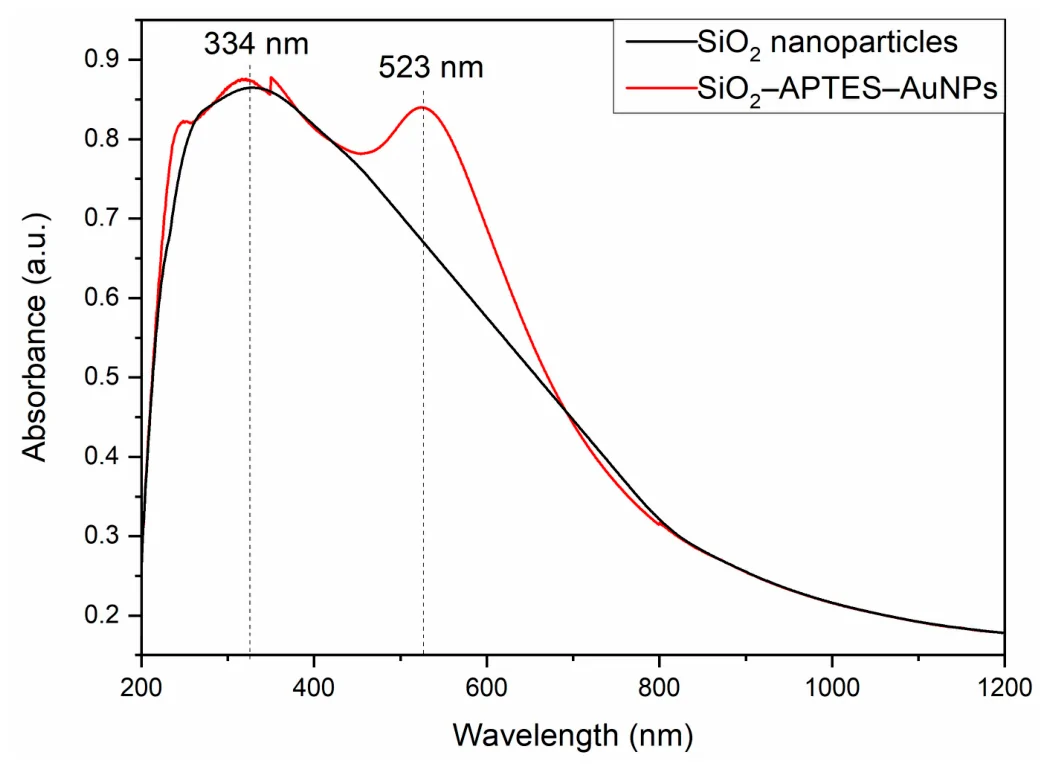
UV–VIS spectra of SiO_2_ nanoparticles and SiO_2_–APTES–AuNPs recorded from 200 to 1200 nm. The SiO_2_ contribution is observed near 330 nm, while the SiO_2_–APTES–AuNP material exhibits an additional plasmonic band near 523 nm.

A Lambda 40 UV–VIS DRS instrument was employed with a solid sample adapter. Spectroscopic analysis was conducted within the range of 200–1200 nm. The maxi-mum absorption at 334 nm is shown in Figure 3, which can be attributed to the formation of nanostructured SiO_2_. This attribution is supported by corresponding bands for silicates, previously reported at approximately 300 nm. This band can be attributed to defects in the SiO_2_ nanoparticles. The probable defects can be Si and O_2_ vacancies or interstitial positions inside the SiO_2_ framework [35].

The reduction and stabilization of gold (Au^3+^ → Au_(s)_) were confirmed through the addition of inorganic ions by UV–VIS analysis of the SiO_2_–APTES–AuNPs. The SiO_2_-APTES with auric hydroxide complex itself did not exhibit the characteristic absorption of gold [14] until gold reduction occurred and metallic clusters formed, giving rise to the characteristic optical response of AuNPs (Figure 3).

The UV–VIS spectrum of the SiO_2_–APTES–AuNPs (Figure 3) shows two prominent bands. The first band, centered at approximately 334 nm, is also observed in the SiO_2_ nanoparticles. The second band, situated at 523 nm, can be attributed to the characteristic plasmonic response associated with gold nanoparticle formation [37].

Although isolated ultrasmall AuNPs may exhibit weak or poorly defined plasmonic responses, the band at 523 nm may be influenced by collective plasmonic coupling between closely spaced AuNPs supported on SiO_2_. Such coupling is favored by the high surface density of AuNPs observed by electron microscopy and by the solid-state reflection measurements conditions [38].

### 3.3. XRD Analysis

**Figure 4:**
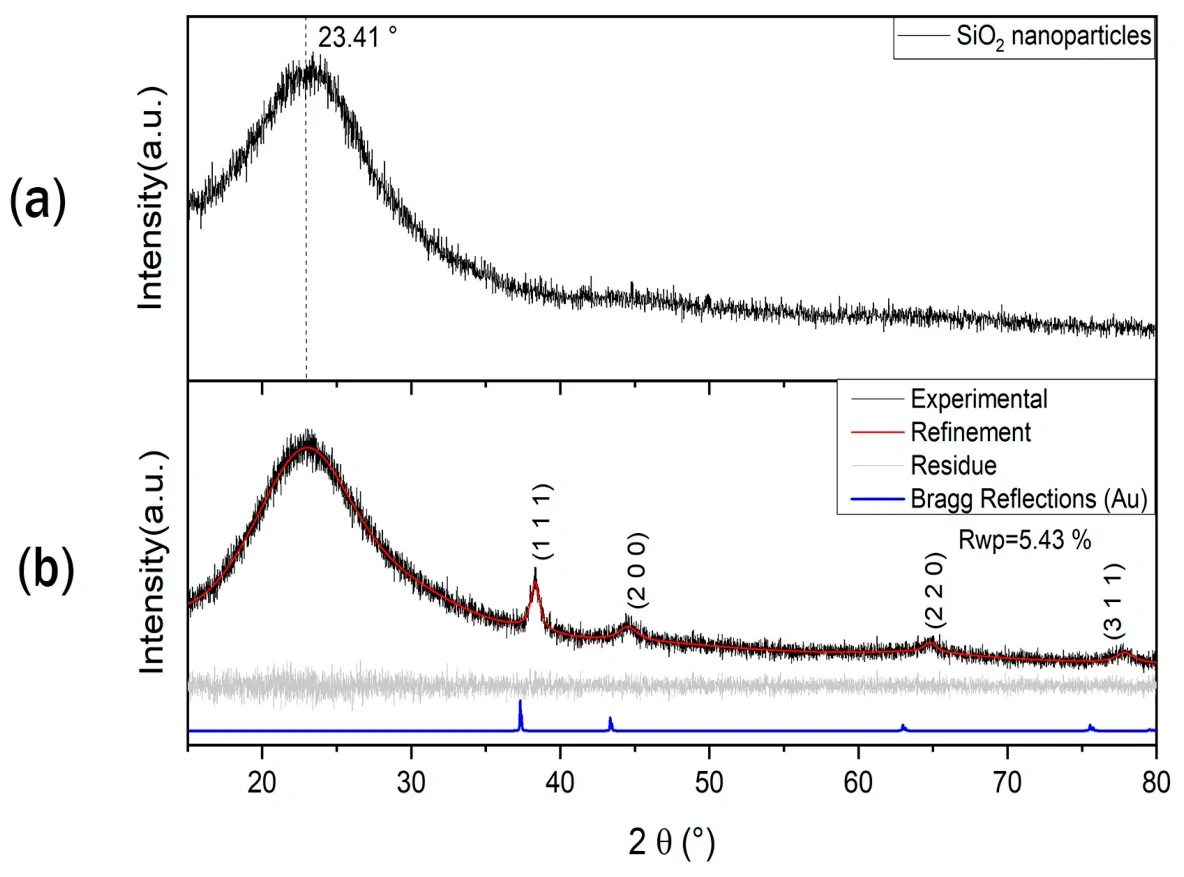
Comparative XRD patterns of (**a**) SiO_2_ nanoparticles and (**b**) SiO_2_–APTES–AuNPs with Rietveld refinement. Patterns were collected over 5–90° in 2θ using Cu Kα radiation (λ = 1.54 Å).

The XRD pattern for the SiO_2_ nanoparticles (Figure 4a) revealed a singular prominent peak at a 2θ value of 23.41°, which is characteristic of predominantly amorphous silica nanoparticles [39]. Amorphous solid lank long-range structural periodicity but retain short-range order, as reflected by the broad scattering feature extending over approximately 10° in 2θ. This behavior is consistent with the characteristic diffraction profile reported for amorphous SiO_2_.

We performed a XRD study of SiO_2_ nanoparticles coated with gold nanoparticles. The XRD pattern (Figure 4b) was subjected to Rietveld refinement to analyze the crystalline structure and estimate the crystallite size of the gold phase. The fit between the experimental and calculated patterns is good, with a reliability factor (Rwp) of 5.43%, indicating a satisfactory quality of refinement.

The SiO_2_–APTES–AuNPs diffraction pattern exhibits Bragg reflections associated with crystalline Au. Rietveld refinement yielded apparent coherent-domain estimates that vary by reflection (11.95, 5.14, 8.98, and 7.03 nm for the reflections analyzed). These XRD-derived values should not be treated as direct particle diameters and, in the present ultrasmall supported-Au system, they differ substantially from the direct TEM particle-size distribution (2.00 ± 0.31 nm). The comparatively well-defined Au reflections may therefore reflect volume-weighted contributions from coherent or partially coalesced Au domains and from a minor fraction of larger crystallites, whereas TEM sizing represents individual AuNP diameters. The discrepancy indicates a limitation of using the present line-profile/refinement estimates as a nanoparticle-size metric; consequently, TEM is used here for direct AuNP sizing, while XRD is used primarily to support identification of crystalline Au and the FCC structure. The refined lattice parameter was 0.4068 nm. This distinction is consistent with literature emphasizing the interpretation limits of X-ray line-profile estimates for polycrystalline gold nanostructures [40].

The presence of well-defined Bragg reflections confirms the crystalline gold coating, whereas the broad reflection under 30° is typical of the amorphous SiO_2_ matrix.

### 3.4. SEM and TEM of SiO_2_ Nanoparticles

**Figure 5:**
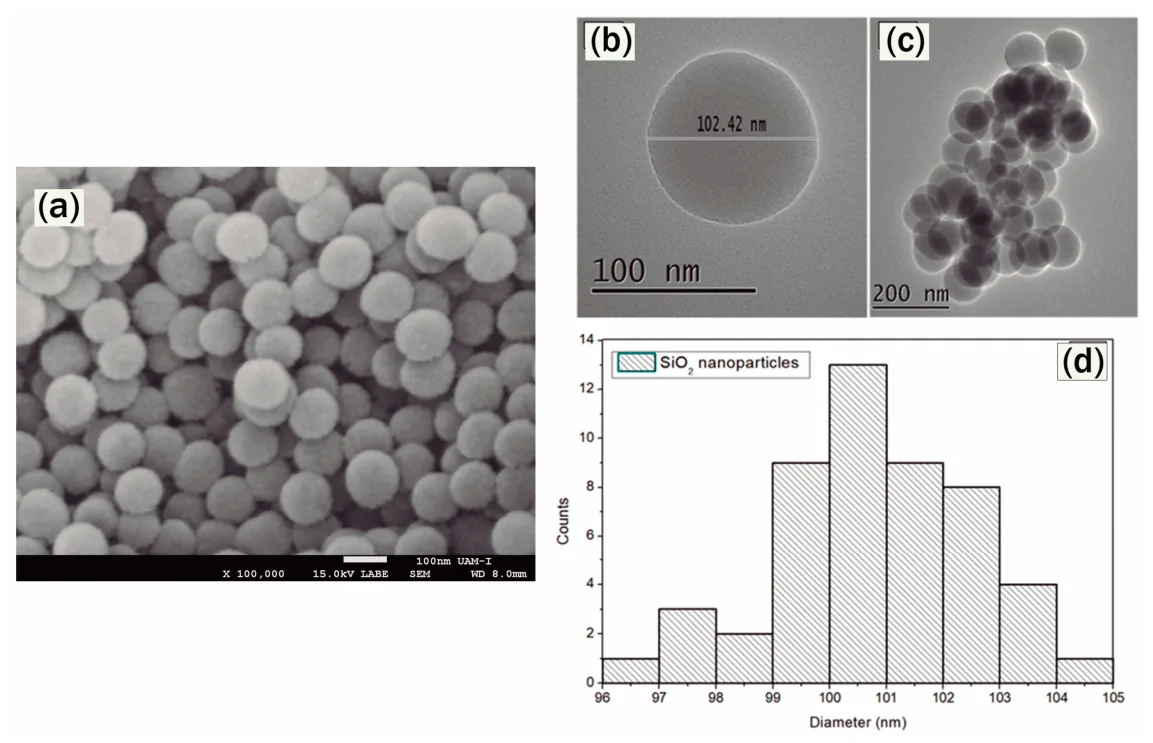
Electron microscopy of SiO_2_ nanoparticles: (**a**) SEM image showing the spherical morphology of the SiO_2_ nanoparticles; (**b**) TEM image of an isolated nanoparticle with its measured diameter; (**c**) TEM image of a nanoparticle cluster; and (**d**) particle-size distribution histogram.

SEM images of the SiO_2_ nanoparticles (Figure 5a) were obtained using a Jeol-SEM 7600F scanning electron microscope, which was configured at 15 kV. SEM images of SiO_2_ reveal a well-defined spherical morphology, which is consistent with the expected outcome of the synthesis method. Notably, the trend toward monodispersity in nanoparticle size should be highlighted.

TEM images were obtained using a Jeol JSM 7600-F instrument (JEOL Ltd., Tokyo, Japan) at a power of 200 kV using the brightfield technique. An isolated nanoparticle representative of the sample is shown in Figure 5b, given that its size falls within the expected range relative to the overall average. Conversely, in Figure 5c, a cluster of nanoparticles is observed, which were considered to generate the size distribution histogram depicted in Figure 5d.

The SiO_2_ particle-size estimate was based on 50 randomly selected, distinct particles from the available TEM micrographs (*n* = 50), yielding an average diameter of 100.81 ± 1.74 nm. This dataset is used to describe the observed narrow particle-size distribution and is not presented as an exhaustive statistical validation of monodispersity.

To identify conditions yielding SiO_2_ nanoparticles with a narrow particle-size distribution, different synthesis conditions were evaluated; the corresponding optimization micrographs are provided in Supplementary Figures S3 and S3.1.

### 3.5. SiO_2_–APTES SEM and TEM

**Figure 6:**
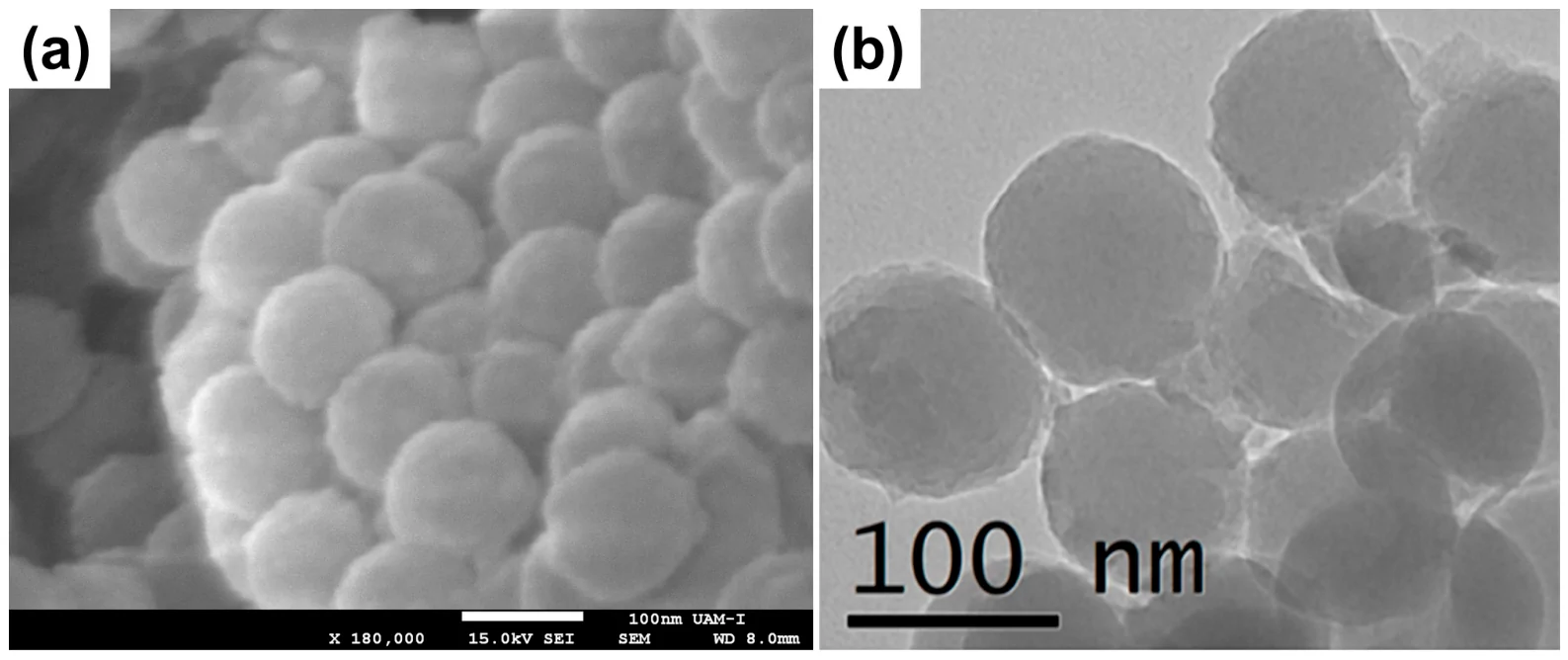
Morphological characterization of SiO_2_–APTES nanoparticles by (**a**) SEM and (**b**) TEM images.

The functionalized nanoparticles were characterized using SEM and TEM to observe changes from a flat to a rough surface and size compared with those of the precursor used in this synthesis step. As shown in Figure 6, the size of the nanoparticles did not significantly change, and a particle diameter of approximately 100 nm was maintained. However, there is a slight difference compared with the previously obtained well-defined spherical shape of the nanoparticles.

As shown in Figure 6a, the SEM image of the functionalized nanoparticles reveals surface features that appear irregular. As shown in Figure 6b, TEM revealed the formation of irregular layers on the surface of the nanoparticles, creating features that altered the surface of the well-defined spheres observed in Figure 5. These changes are attributed to the formation of surface layers composed of APTES coating, indicating the successful functionalization reaction with the amino groups on the surface of the SiO_2_ nanoparticles. Representative TEM micrographs of the SiO_2_–APTES optimization samples are provided in Supplementary Figure S4.

### 3.6. SEM and TEM Characterization of SiO_2_–APTES–AuNPs

**Figure 7:**
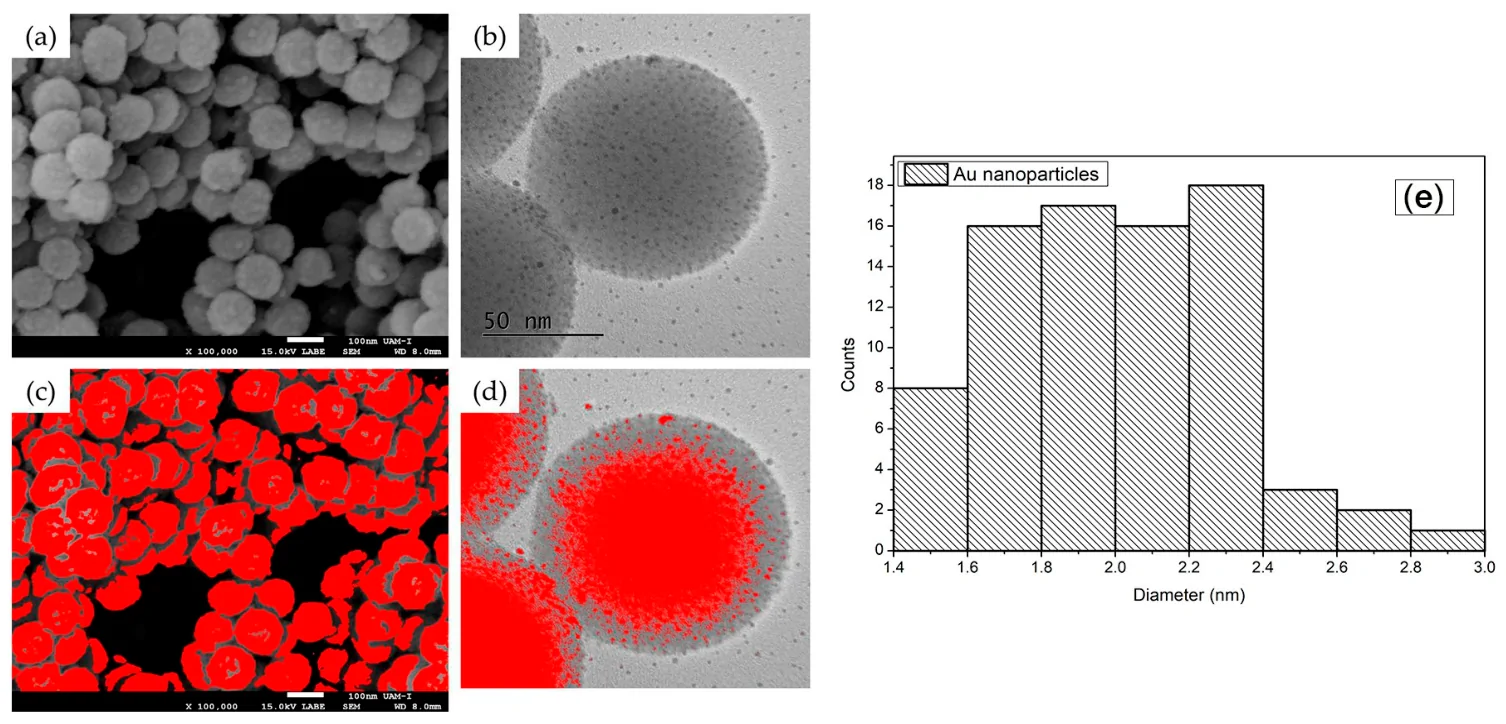
Microscopic images of the gold coating of SiO_2_-APTES-AuNPs. (**a**) SEM image of the sample, taken at ×100,000 magnification. (**b**) TEM image of the sample. (**c**) Segmentation result of AuNPs in the SEM image. (**d**) Segmentation of AuNPs in the TEM image. (**e**) Size distribution histogram of the supported AuNPs. A total of 80 AuNPs were measured (*n* = 80), yielding an average diameter of 2.00 ± 0.31 nm.

SEM and TEM images of the SiO_2_–APTES–AuNPs are shown in Figure 7. As shown in Figure 7a, the SEM image of the sample reveals the presence of clusters of SiO_2_–APTES–AuNPs, as shown in Figure 7b, the TEM images of the sample disclose that the size and morphology of the SiO_2_ matrix did not change. Additionally, a uniform coating of AuNPs was observed; however, there was evidence of gold nanoparticles not attached to the support, indicating a significant excess of the gold reagent used or detachment of gold nanoparticles from the support during some part of the procedure. In this regard, other conditions were tested, and different sizes of AuNPs were observed (Supplementary Figure S5). Additional characterization of the SiO_2_ nanoparticles is provided in Supplementary Figure S6 (thermogravimetric analysis, TGA) and Supplementary Figure S7 (XRD pattern of SiO_2_ nanoparticles).

In this case, we used the segmentation method to identify the region of interest thresholding to identify gold nanoparticles. We applied an indexed image with a possibility of 256 shades of gray where 0 is black and 255 is white. In Figure 7c, the nanoparticles of interest are graded from gray to white according to a threshold of gray tones, and the gold particles in Figure 7d are taken from black to white. The images were processed in Fiji (ImageJ 1.54p, National Institutes of Health, USA). This image analysis describes the AuNPs features visible in the analyzed micrographs and should not be interpreted as a whole-substrate Raman-uniformity measurement.

Particle detection was then performed using the Analyze Particles function, with a minimum size threshold of 5 pixels^2^ (5–Infinity), excluding sub-resolution noise artifacts while retaining nanoparticle signals regardless of upper size limit. The resulting binary particle map was overlaid onto the original grayscale SEM image for visual validation, as shown in Figure 7c,d. Spurious detections arising from residual background noise, edge artifacts, or agglomerate misclassification were manually curated and removed. Validated particle outlines were highlighted in red to allow direct visual correspondence between segmented regions and the underlying nanoparticle morphology in the original micrograph.

From Figure 7b, we conducted random measurements of the diameters of the AuNPs supported on the SiO_2_-APTES surface to generate the size distribution histogram presented in Figure 7e, revealing the prevalence of nanoparticles with sizes ranging from 1.6 to 2.4 nm. With a total of 80 measurements considered, the mean with its respective standard deviation was obtained, yielding an average AuNP diameter of 2.00 ± 0.31 nm.

### 3.7. Raman Response

**Figure 8:**
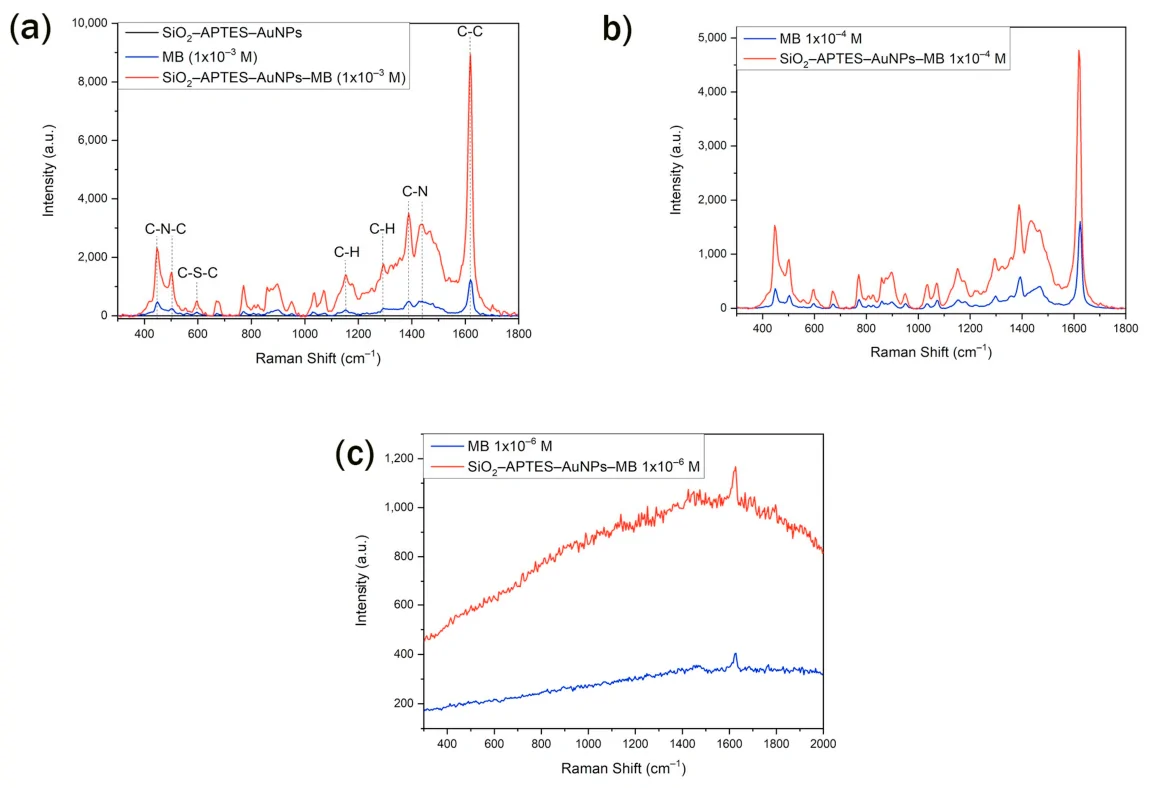
Raman spectra of MB at (**a**) 1 × 10^−3^ M, (**b**) 1 × 10^−4^ M, and (**c**) 1 × 10^−6^ M, recorded in the presence and absence of the SiO_2_-APTES-AuNPs substrate using 532 nm excitation, 15 µW incident power at the sample surface, a 50× objective (NA 0.75), 4 s integration time, and 10 accumulations. Samples were prepared by drop-casting and thermal drying as described in Section 2.5. Spectra in panels (**a**,**b**) were baseline-corrected using airPLS, whereas no baseline correction was applied to the spectra in panel (**c**) to preserve the fluorescence-like background; no additional spectral processing was applied. Red and blue spectra correspond to MB deposited on the SiO_2_-APTES-AuNPs substrate and MB without the substrate, respectively; the black spectrum in panel (**a**) corresponds to the SiO_2_-APTES-AuNPs substrate alone.

Raman spectra of the SiO_2_–APTES–AuNP substrate and MB were recorded. As shown in Figure 8a, no characteristic Raman bands attributable to the SiO_2_–APTES–AuNPs substrate were observed within the investigated spectral range. In contrast, MB exhibited its characteristic vibrational bands, with the most prominent band at 1620 cm^−1^, assigned to C–C ring stretching. Other significant bands appeared at 1433 and 1390 cm^−1^, corresponding to asymmetric and symmetric C–N stretching vibrations, respectively, associated with the dimethylamine groups. Several C–H bending modes were also observed, including the in-plane ring deformation at 1297 cm^−1^, in-plane vibrations at 1153, 1029, 952, 900, and 773 cm^−1^, and an out-of-plane bending mode at 670 cm^−1^. Skeletal deformation modes of C–S–C at 598 cm^−1^ and C–N–C at 503 and 448 cm^−1^ were also identified [20].

To investigate the enhancement potential of the SiO_2_–APTES–AuNP substrate, MB was used as a molecular probe at three different concentrations (10^−3^, 10^−4^, and 10^−6^ M). Raman spectra of MB were recorded in the presence and absence of the SiO_2_–APTES–AuNPs substrate.

When evaluating the response of the SiO_2_–APTES–AuNPs substrate at different MB concentrations (Figure 8), concentration-dependent differences were observed. At 10^−3^ and 10^−4^ M, similar Raman spectral profiles were obtained for MB in the presence and absence of the SiO_2_–APTES–AuNP substrate. At these concentrations, conventional Raman scattering from bulk or unadsorbed MB may contribute substantially to the measured signal and may partially mask any surface-enhanced contribution. Therefore, similarity between the spectral profiles should not by itself be interpreted as evidence for a specific SERS enhancement mechanism. The measurements are treated here as a preliminary spectroscopic evaluation rather than a quantitative validation of SERS performance.

The relative signal amplification was evaluated using the integrated area of the Raman band at approximately 1620 cm^−1^ [17]. However, MB was not homogeneously distributed over the substrate surface, which is consistent with the drop-casting preparation method. During drying, solvent evaporation induces capillary flows that transport the solute from the center of the droplet toward its edges, producing the so-called coffee-ring effect [40]. Due to the non-uniform distribution of the analyte inherent to this preparation method, a quantitative SERS enhancement factor was not determined. Instead, relative intensity ratios were used as a preliminary qualitative assessment of signal amplification [17]. Using the integrated area of the band at approximately 1620 cm^−1^, relative signal amplification ratios of approximately 7.5 and 3.5 were obtained for MB concentrations of 10^−3^ and 10^−4^ M, respectively (Figure 8a,b).

At a MB concentration of 10^−6^ M, a dominant fluorescence-like background was observed in the Raman spectra (Figure 8c). The background increased in the presence of the SiO_2_–APTES–AuNPs substrate and partially obscured the Raman features. Because direct fluorescence spectrum, fluorescence lifetimes, and quantum-yield measurements were not acquired, this observation does not establish metal-enhanced fluorescence (MEF) or the coexistence of SERS and MEF. It is therefore interpreted only as a fluorescence-like contribution that warrants dedicated fluorescence measurements in future work.

Previous studies have shown that fluorophore–metal distance can influence fluorescence quenching or enhancement near plasmonic surfaces. For example, SiO_2_ dielectric spacers have been used to control separation between fluorophores and nanoporous gold, with reduced quenching reported at selected spacer distances. This literature provides a possible mechanistic context for future experiments, but it does not demonstrate that MEF occurs in the present SiO_2_–APTES–AuNP-MB system.

The measured response may contain contributions from several processes, including conventional Raman scattering, surface-mediated Raman enhancement, fluorescence background, and concentration-dependent MB aggregation. The relative contribution of these processes cannot be quantitatively separated from the present dataset, particularly because the analyte distribution produced by drop-casting is non-uniform.

Accordingly, the increased background observed at 10^−6^ M is described as fluorescence-like rather than as evidence that metal-enhanced fluorescence predominates over Raman enhancement.

These proof-of-concept observations motivate further characterization of the SiO_2_–APTES–AuNP material. A rigorous assessment should include controlled analyte deposition, Raman mapping, spot-to-spot and batch-to-batch statistics, conventional SERS enhancement-factor determination where appropriate, and direct fluorescence measurements such as emission spectra and, ideally, lifetime or quantum-yield measurements.

### 3.8. Theoretical Results

DFT calculations were used to examine qualitative local interactions between MB and the Au surface. The Au_32_ model (its diameter is approximately 9 nm) is smaller than the approximately 2 nm AuNPs measured by TEM and does not include the SiO_2_-APTES support; consequently, it cannot reproduce the size-dependent electronic/plasmonic properties of the complete experimental material. Its role is limited to identifying plausible local adsorption motifs and contacts that may influence molecular orientation and vibrational response. Methodological studies combining SERS, DFT-calculated Raman spectra, and independent analytical identification provide a useful example of the level of complementary evidence required for stronger spectral assignments [41].

**Figure 9:**
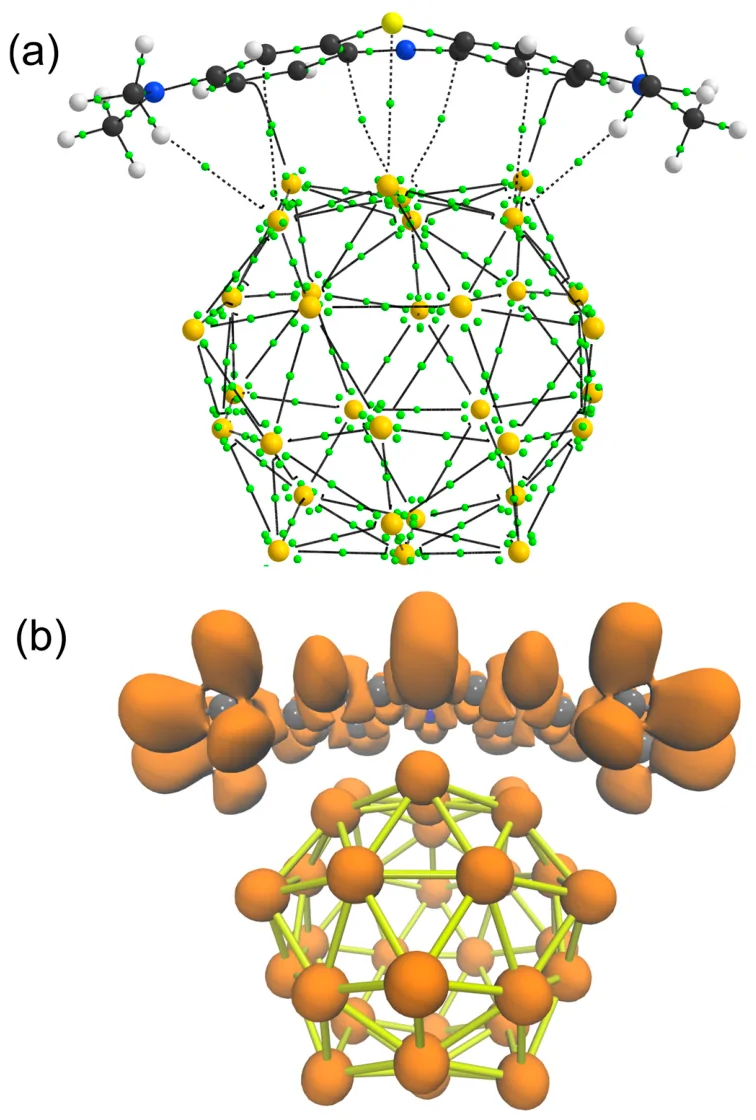
(**a**) Bond critical points (green dots) and bond paths (black lines), and (**b**) electron localization function (0.7 isosurface) of the most stable AuNP-MB complex.

The first observation is that the PBE0-optimized structures differ only slightly from the r2SCAN-3c ones, which highlights the ability of this composite method to obtain accurate geometries at lower cost. The most important result from the geometrical analysis is that all the perpendicular configurations eventually reach a planar configuration after the optimization process. This was corroborated by repeating the geometry optimizations directly with the DFT methodology to discard the possibility that these results were biased by r2SCAN-3c. All the initial configurations lead to 3 different AuNP-MB complexes. In three of them, the MB molecules bend toward the AuNPs, confirming the strong affinity of these nanoparticles for methylene blue. In the most stable complex, the sulfur atom interacts with one gold atom at 3.412 Å. A corresponding bond critical point (BCP) and bond path, which in QTAIM is indicative of the presence of an atom–atom interaction, are observed in Figure 9a. The value of the Laplacian of the electron density at this BCP is positive, which is indicative of a non-covalent nature. This is corroborated by the absence of a disynaptic basin (orange sur-faces in Figure 9b) between the Au and S atoms in the ELF function (such as those found in the bonded atoms of MB, which in this theory exist whenever two atoms are connected covalently). Indeed, no disynaptic basis was found between any of the AuNP and MB atoms. Thus, it can be concluded that MB is adsorbed by physisorption.

**Figure 10:**
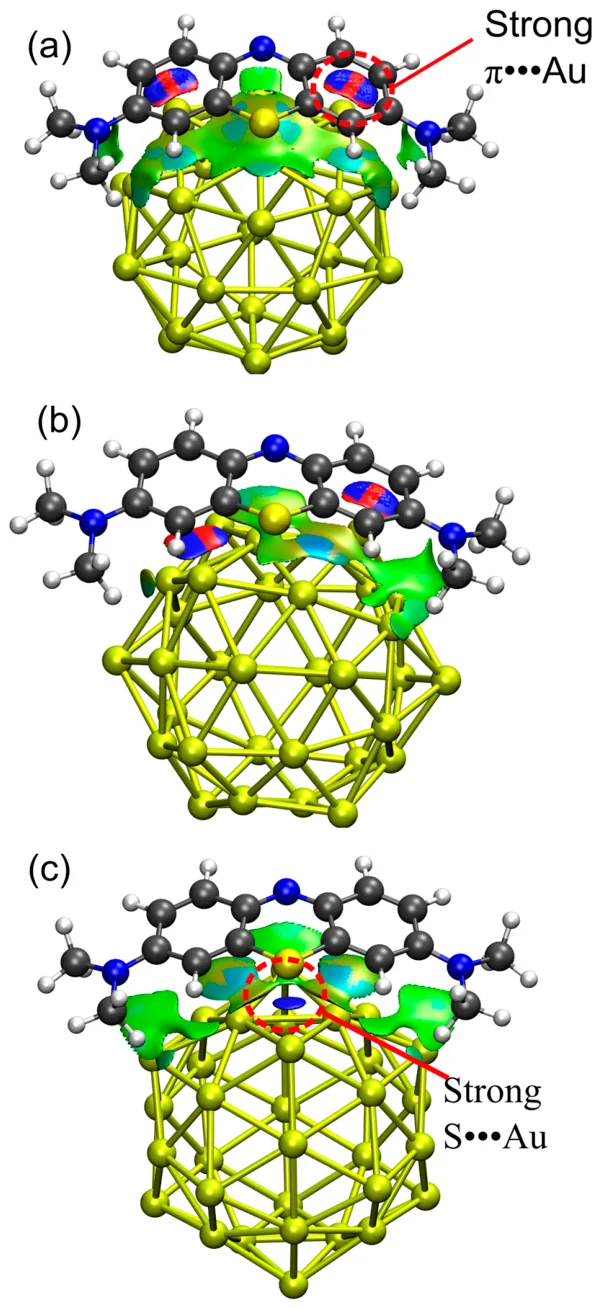
NCI isosurfaces (0.3) of the (**a**) first, (**b**) second and (**c**) third-most stable AuNP-MB complexes studied in this work.

In addition to the S∙∙∙Au interactions∙∙∙, some C-H∙∙∙Au contacts can be observed in Figure 9, which contribute to stabilizing the AuNP-MB system. Furthermore, a deeper understanding of the forces behind this complex can be obtained from the NCI isosurface analysis, which is shown for the most stable AuNP-MB structure in Figure 10a. The large flat green surface indicates the regions where van der Waals forces are present. These interactions play a predominant role, even in regions where S∙∙∙Au and C-H∙∙∙Au interactions are found. Interestingly, very strong interactions occur between the outer aromatic rings of MB and the AuNPs, particularly with the two closer carbon atoms of each ring, which in NCI are observed as blue regions that imply some degree of localized interactions.

The following stable AuNP-MB complex, which is 4.7 kcal/mol higher in energy, also shows π∙∙∙Au interactions, but the S∙∙∙Au complex is missing (Figure 10b). On the other hand, the least stable AuNP-MB complex (8.1 kcal/with respect to the most stable complex) has a very strong and localized S∙∙∙Au interaction (2.623 Å), which is observed as a blue disk-shaped surface in the NCI isosurface (Figure 10c). Thus, the analysis of these 3 structures reveals that both S∙∙∙Au and π∙∙∙Au interactions are the most relevant specific forces responsible for AuNP-MB complex formation, along with delocalized van der Waals interactions. These results are consistent with those of other studies on the adsorption of aromatic compounds on gold nanoparticles [18,23,28].

The following section describes the changes observed in the Raman spectra calculated with r2-SCAN-3c, which can serve as a guide for the quantitative exploration of a SERS effect of this molecule in future work. Although the cluster of 32 gold atoms is not yet large enough to represent a 2 nm nanoparticle, and the SiO_2_–APTES support is absent in the present model, the use of this type of clusters (of equal or smaller size) is common practice because it accurately reflects local analyte-NP interactions and provides at least a qualitatively accurate description of the expected effects in a SERS phenomenon [27,42,43,44]. Because these calculations do not consider the electromagnetic mechanism and resonance effects, the present analysis will focus on the changes observed due to the contribution of the chemical mechanism. To evaluate this in the ground state and eliminate the effects of thermal coupling or mass-dependent kinetic factors at the correlated frequencies, the calculated Raman activities were analyzed directly instead of the corrected intensities. The observed modifications in the activities directly reflect the intrinsic perturbation of the molecular polarizability tensors due to static charge transfer between Au_32_ and MB. This analysis was conducted on the most stable AuNP-MB complex.

The first thing to notice is that the highest peak in the MB spectrum (Supplementary Figure S8), at 3012 cm^−1^ (which is made up of the combination of symmetric and asymmetric C-H stretching), splits into two intense peaks; one is redshifted (2958 cm^−1^) and the other is blue shifted (3067 cm^−1^). This is caused by a break in the symmetry of the molecule, as some hydrogens interact directly with the nanoparticle, as discussed earlier in the QTAIM analysis (giving a redshifted signal), while those that are freely stretched in the opposite direction give the blue shifted signal.

The peak corresponding to the stretching of the C-C bonds of the aromatic rings (1640 cm^−1^) is also redshifted to 1599 cm^−1^. This is a consequence of the previously mentioned π∙∙∙Au interactions, which involve a charge transfer from MB to AuNP (approximately 0.2 electrons according to Mulliken charges). Furthermore, an increase in activity is observed in the AuNP-MB complex, corroborating that the chemical mechanism contributes to the SERS phenomenon. This can also be corroborated by analyzing the C-C bond distances, which in the AuNP-MB complex experience elongations of between 0.002 and 0.032 Å.

On the other hand, the peak at 1412 cm^−1^, attributed to the symmetrical C–N stretching vibrations of the phenothiazine ring coupled with in-plane C–H bending modes, undergoes a slight blueshift at 1426 cm^−1^, indicating that the charge redistribution implies a slight strengthening of the bonds involved. This peak also shows an increase in the Raman activity in the complex. Finally, the peaks associated with C-N-C deformation at 446 and 503 cm^−1^ do not undergo any significant displacement, although an increase in Raman activity is also observed in the complex, particularly for the second signal.

## 4. Conclusions

SiO_2_–APTES–AuNPs nanostructures were successfully prepared through a stepwise route involving SiO_2_ nanoparticles synthesis, SiO_2_-APTES surface functionalization, and gold deposition using an inorganic stabilization strategy for the AuNPs without conventional organic AuNP capping agents. FTIR, UV-VIS, XRD, SEM, and TEM collectively support formation of hybrid material. TEM yielded average diameters of 100.81 ± 1.74 nm for SiO_2_ nanoparticles (*n* = 50) and 2.00 ± 0.31 nm for supported AuNPs (*n* = 80).

The Raman evaluation of the SiO_2_–APTES–AuNPs substrate provides proof-of-concept evidence of concentration-dependent changes in the measured MB response. Relative Raman signal-amplification ratios were observed at 10^−3^ and 10^−4^ M, but these values should not be interpreted as conventional SERS enhancement factors because the drop-cast MB distribution was non-uniform. At 10^−6^ M, an increased fluorescence-like background was observed; without direct fluorescence measurements, this result does not demonstrate MEF or a dual Raman/fluorescence sensing response. The non-uniform analyte distribution remains an important limitation for quantitative assessment of substrate performance.

The Au_32_-based theoretical analysis provides qualitative molecular-level insight into local MB-Au interactions, with S···Au and π···Au contacts contributing to stabilization of the modeled complexes. Because the model does not reproduce the experimental AuNP size or include the SiO_2_-APTES support, these calculations are interpreted as local interaction models rather than quantitative representations of the complete nanostructure.

Overall, the study establishes a synthesis and physicochemical characterization route for SiO_2_-supported AuNPs using an inorganic stabilization strategy for the gold phase and provides preliminary evidence supporting further Raman-based investigation of the material. Comprehensive Raman/SERS mapping, spot-to-spot and batch-to-batch reproducibility studies, controlled analyte deposition, and direct fluorescence measurements are required before quantitative sensing performance or fluorescence-enhancement mechanisms can be established.

## Data Availability

The datasets used and/or analyzed during the current study are available from the corresponding author on reasonable request.

## Supplementary Materials

The following supporting information can be downloaded at: https://www.mdpi.com/article/10.3390/nano16191243/s1, Figure S1: FTIR spectra of SiO_2_ nanoparticles prepared under the different synthesis conditions evaluated in this study; Figure S2: FTIR spectra of SiO_2_-APTES nanoparticles prepared under the different functionalization conditions evaluated in this study; Figure S3: TEM micrographs of SiO_2_ nanoparticles: (a) Sample 1, (b) Sample 2, and (c) Sample 3; Figure S3.1. TEM micrographs of SiO_2_ nanoparticles: (a) Sample 4, (b) Sample 5, and (c) Sample 6; Figure S4: TEM micrographs of SiO_2_-APTES nanoparticles: (a) Sample 1, (b) Sample 2, and (c) Sample 3; Figure S5: TEM micrographs of SiO_2_–APTES–AuNPs nanostructures: (a) Sample 1, (b) Sample 2, and (c) Sample 3; Figure S6: TGA of SiO_2_ nanoparticles; Figure S7. XRD pattern of SiO_2_ nanoparticles. Figure S8. Raman spectra (constructed with DFT computed normalized activities) of MB (blue) and AuNP-MB (orange).

## Abbreviations

The following abbreviations are used in this manuscript:

airPLS	Adaptive iteratively reweighted penalized least squares
APTES	(3-Aminopropyl)triethoxysilane
AuNPs	Gold nanoparticles
DFT	Density functional theory
FCC	Face-centered cubic
FTIR	Fourier-transform infrared spectroscopy
HAuCl_4_	Tetrachloroauric acid
JEOL	Japan Electron Optics Laboratory
LSPR	Localized surface plasmon resonance
MB	Methylene blue
MEF	Metal-enhanced fluorescence
NCI	Noncovalent interaction index
NH_4_OH	Ammonium hydroxide
QTAIM	Quantum theory of atoms in molecules
SEM	Scanning electron microscopy
SERS	Surface-enhanced Raman scattering
SiO_2_	Silicon dioxide
TEM	Transmission electron microscopy
TEOS	Tetraethyl orthosilicate
TGA	Thermogravimetric analysis
UV-VIS	Ultraviolet–visible spectroscopy
XRD	X-ray diffraction
