# Supplementary material for: Inorganic-Ion-Stabilized Au Nanoparticles Supported on SiO2: Synthesis, Physicochemical Characterization, and Proof-of-Concept Raman Response Toward Methylene Blue
Source: Nanomaterials (Basel). 2026 Oct 1;16(19):1243. doi: 10.3390/nano16191243 (PMC13667054; doi:10.3390/nano16191243)
Supplement: Supplementary file 1 [file nanomaterials-16-01243-s001.zip › nanomaterials-4520341-supplementary.pdf]

Inorganic-Ion-Stabilized Au Nanoparticles Supported on SiO2: Synthesis, Physicochemical Characterization, and Proof-of-Concept Raman Response Toward Methylene Blue

Jorge Luis Mendoza Tellez [1], Omar E. Uribe-Juárez [2], Bruno Landeros-Rivera [3], Alba Adriana Vallejo-Cardona [4], Diana M. Osorio-Londoño [5,6], Federico González García [7], Francisco Tzompantzi [1], Raúl Pérez-Hernández [8], Rebeca Sosa Fonseca [2], Luis Alberto Moreno Ruiz [9], Ana María Soto-Estada [1,*] and Iris N. Serratos [1,*]

[1] Departamento de Química, Universidad Autónoma Metropolitana-Iztapalapa, Av. San Rafael Atlixco 186, Col. Vicentina, México City 09340, Mexico; cbi2173048089@izt.uam.mx (J.L.M.T.); fjtz@xanum.uam.mx (F.T.)

[2] Departamento de Física, Universidad Autónoma Metropolitana-Iztapalapa, Av. San Rafael Atlixco 186, Col. Vicentina, México City 09340, Mexico; ouribe@xanum.uam.mx (O.E.U.-J.); rebe@xanum.uam.mx (R.S.F.)

[3] Departamento de Química Inorgánica, Facultad de Química, Universidad Nacional Autónoma de México, Ciudad Universitaria, Coyoacán, México City 04510, Mexico; brunolanderos@quimica.unam.mx

[4] Biotecnología Médica y Farmacéutica, Centro de Investigación y Asistencia en Tecnología y Diseño del Estado de Jalisco, Av. Normalistas 800, Colinas de La Normal, Guadalajara 44270, Mexico; avallejo@ciatej.mx

[5] Departamento de Ingeniería Eléctrica, Universidad Autónoma Metropolitana-Iztapalapa, Av. San Rafael Atlixco 186, Col. Vicentina, México City 09340, Mexico; dmol@xanum.uam.mx

[6] División de Ingeniería Mecánica e Industrial, Facultad de ingeniería, Universidad Nacional Autónoma de México, Ciudad Universitaria, Coyoacán, México City 04510, Mexico

[7] Departamento de Ingeniería de Procesos e Hidráulica, Universidad Autónoma Metropolitana,
México City 04510, Mexico; fgonzalez@izt.uam.mx

[8] Instituto Nacional de Investigaciones Nucleares, Car. Méx.-Tol. s/n, La Marquesa, Ocoyoacac 52750, Mexico; raul.perez@inin.gob.mx

[9] Centro de Nanociencias y Micro y Nanotecnologías (CNMN) del Instituto Politécnico Nacional, Av. Luis Enrique Erro s/n, Nueva Industrial Vallejo, Gustavo A. Madero, México City 07700, Mexico;
lmorenor@ipn.mx

* Correspondence: amse@xanum.uam.mx (A.M.S.-E.); insa@xanum.uam.mx (I.N.S.)

1. **Fourier-transform infrared (FTIR) spectra of SiO_2 nanoparticles obtained under different synthesis conditions.**

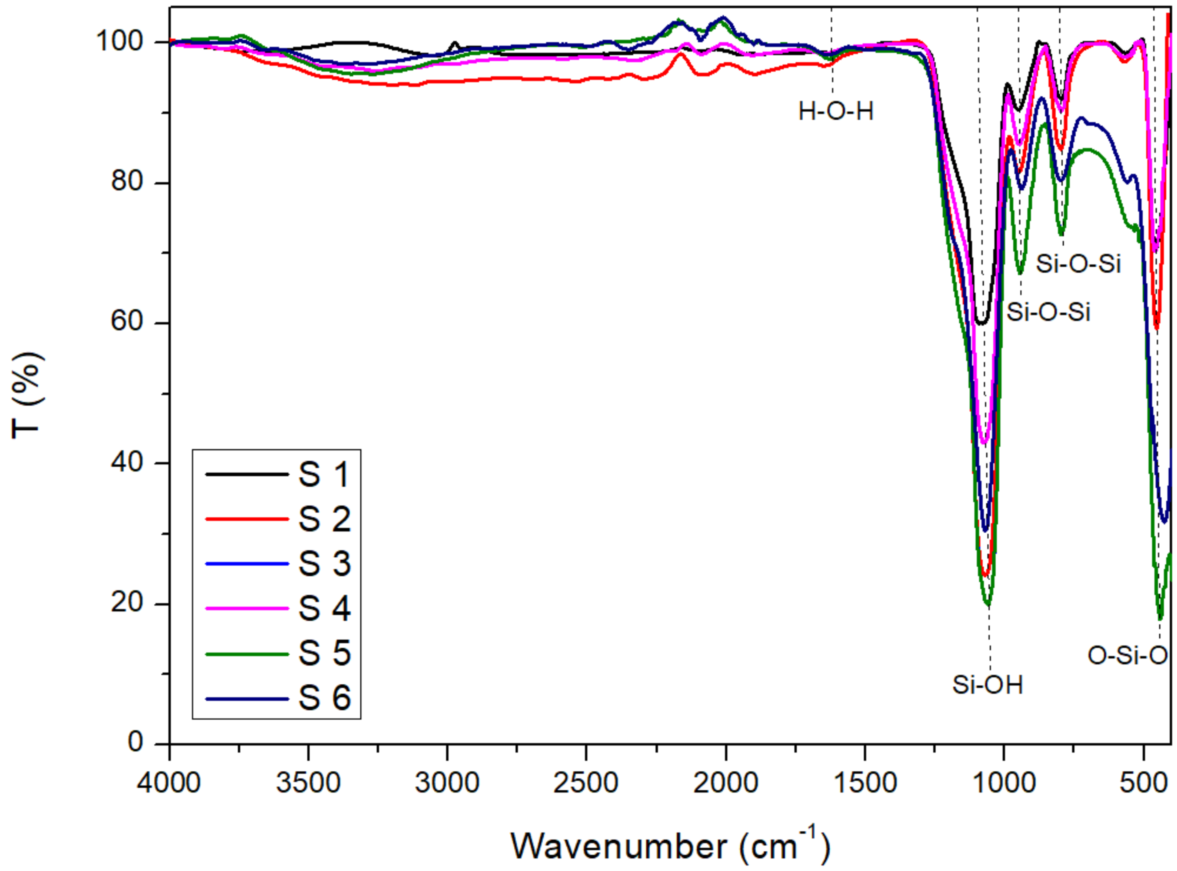

Figure S1. FTIR spectra of SiO_2 nanoparticles prepared under the different synthesis conditions evaluated in this study.

The spectra correspond to SiO_2 samples S1-S6 prepared under the optimization conditions evaluated in this study. The same characteristic Si-O-related bands were observed across the samples.

2. FTIR spectra of SiO_2-APTES nanoparticles obtained under different functionalization conditions.

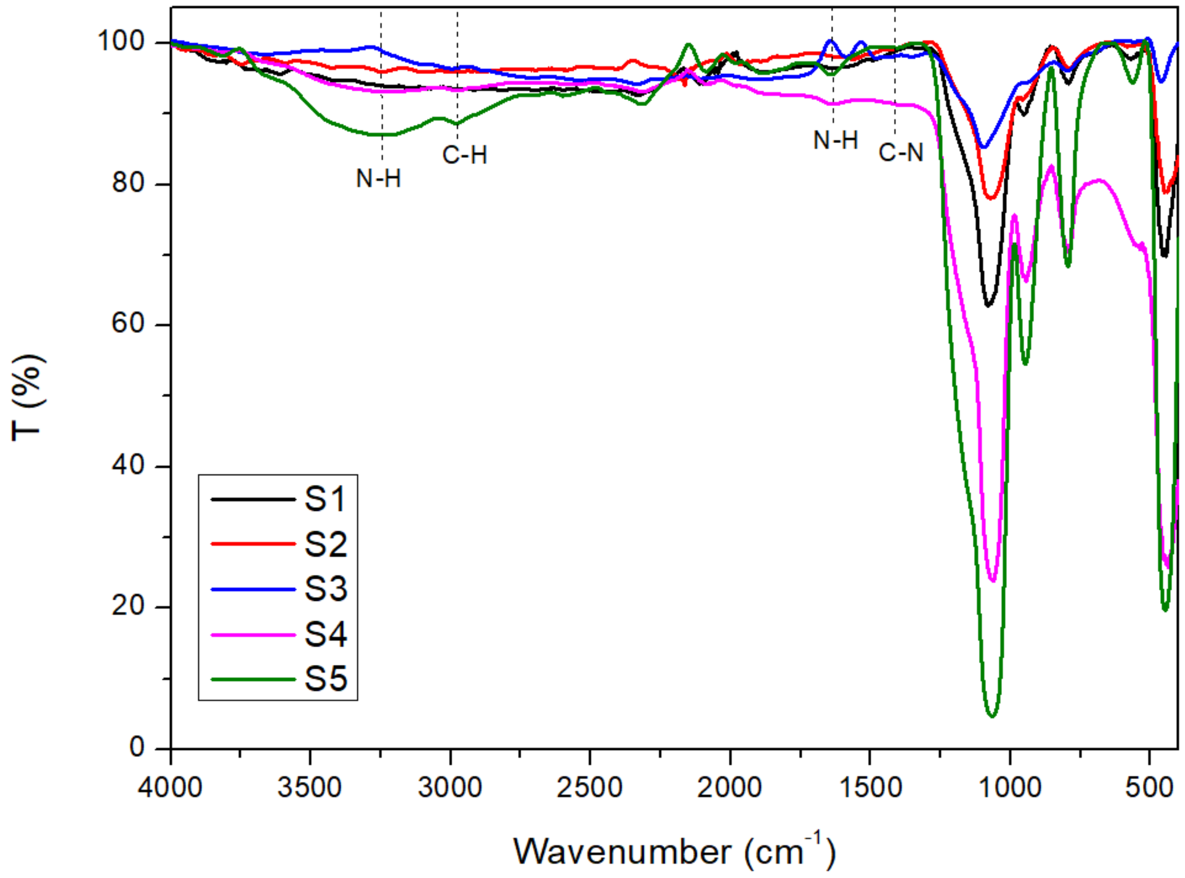

Figure S2. FTIR spectra of SiO_2-APTES nanoparticles prepared under the different functionalization conditions evaluated in this study.

The spectra correspond to SiO_2-APTES samples S1-S5 prepared under the functionalization conditions evaluated in this study. The selected condition showed the characteristic bands assigned in the manuscript to the amine-containing APTES-functionalized surface.

3. Transmission electron microscopy (TEM) of SiO_2 nanoparticles.

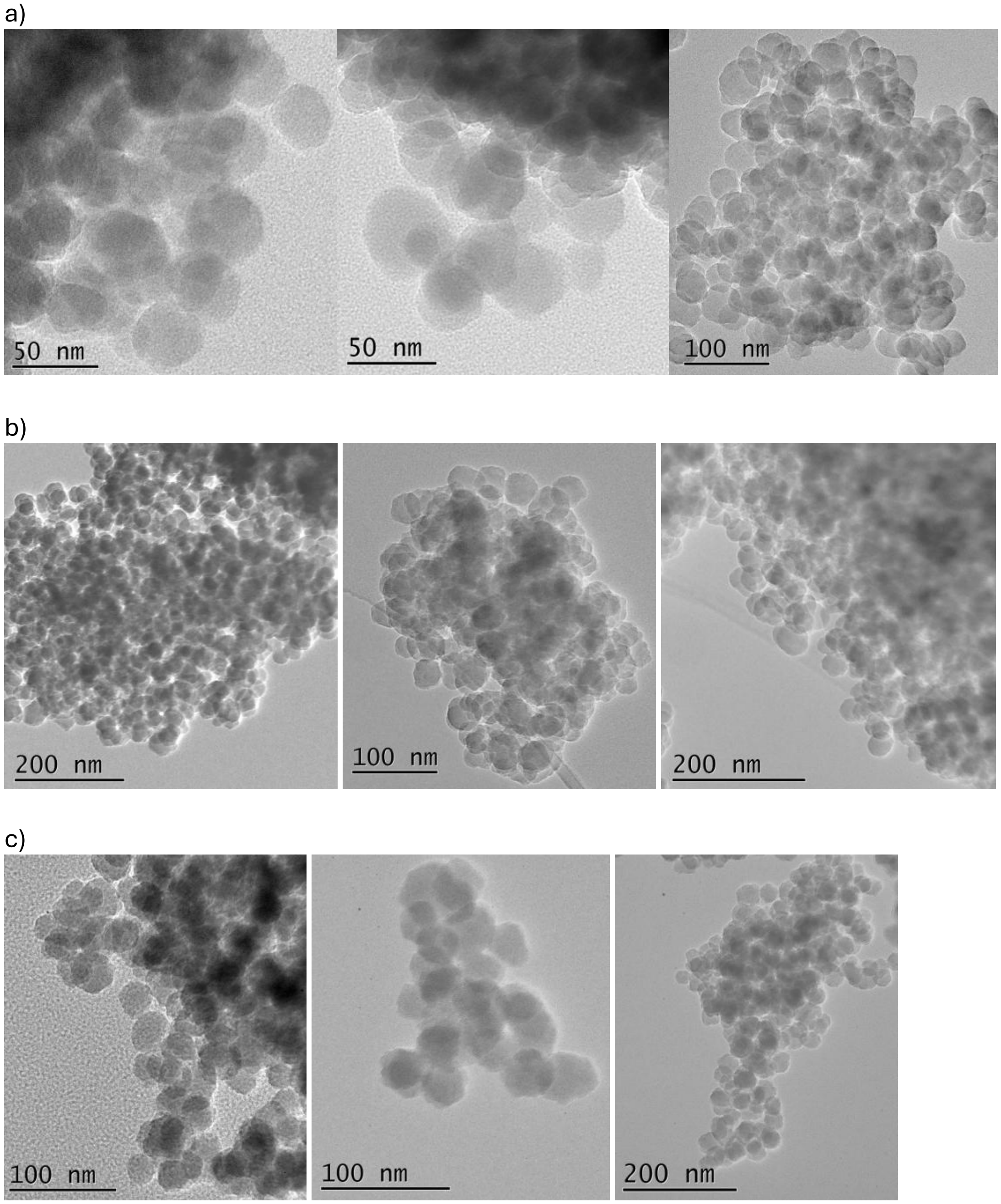

Figure S3. TEM micrographs of SiO_2 nanoparticles: a) Sample 1, b) Sample 2, and c) Sample 3.

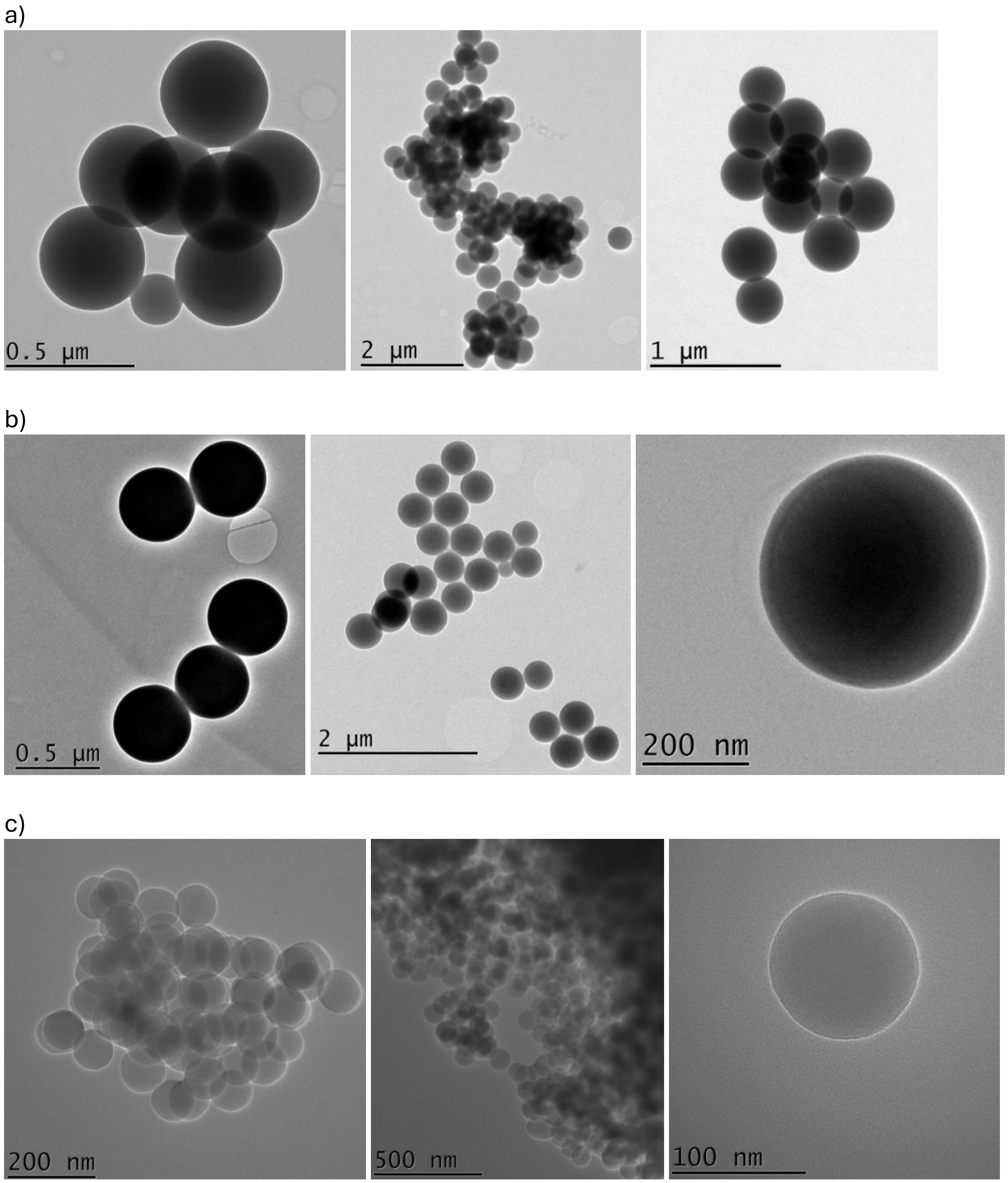

Figure S3.1. TEM micrographs of SiO_2 nanoparticles: a) Sample 4, b) Sample 5, and c) Sample 6.

4. TEM of SiO_2-APTES nanoparticles.

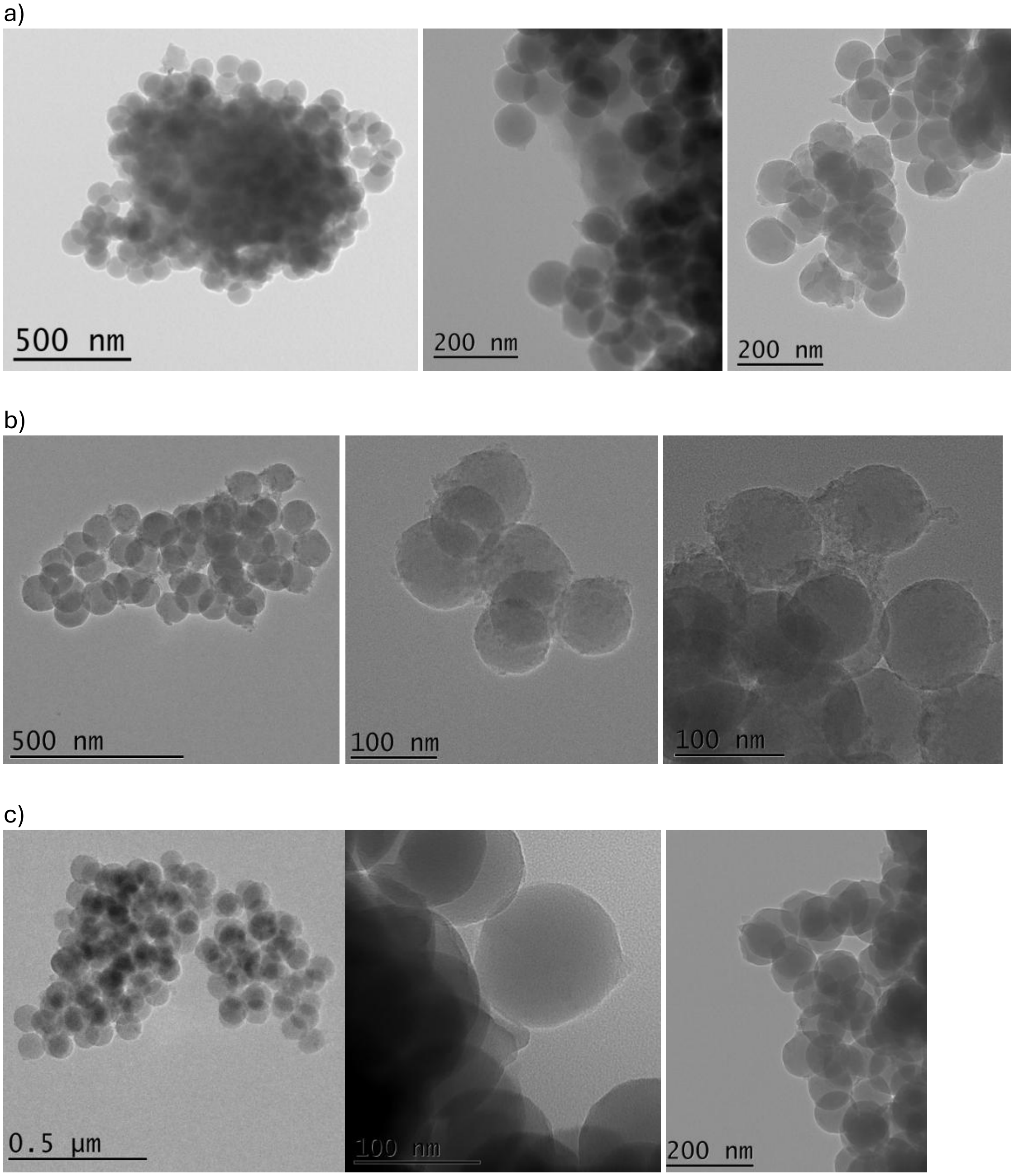

Figure S4. TEM micrographs of SiO_2-APTES nanoparticles: a) Sample 1, b) Sample 2, and c) Sample 3.

5. TEM of SiO_2-APTES-AuNPs nanostructures.

a)

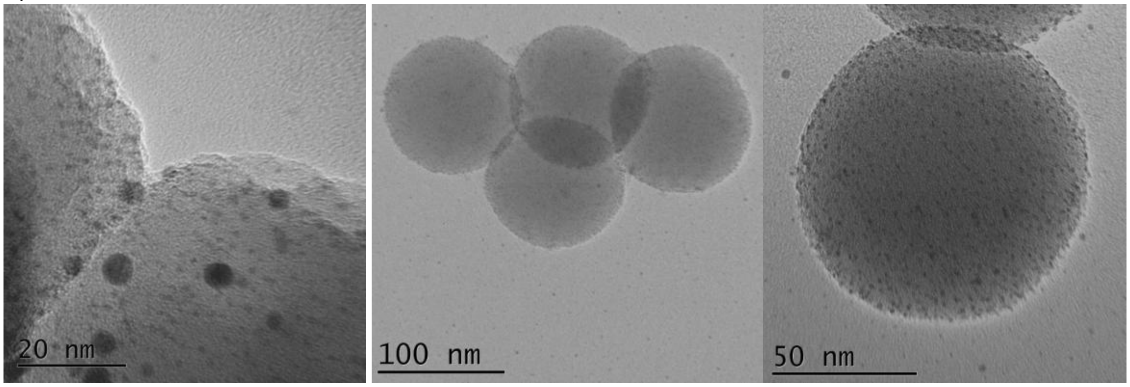

b)

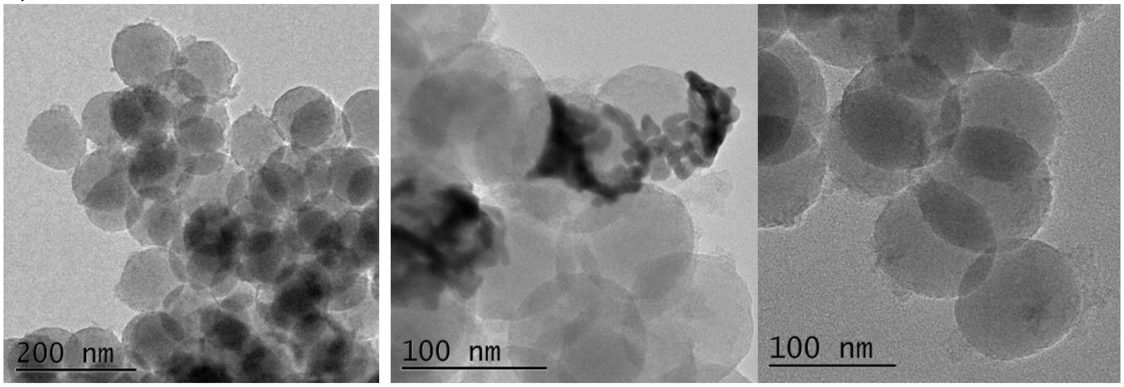

c)

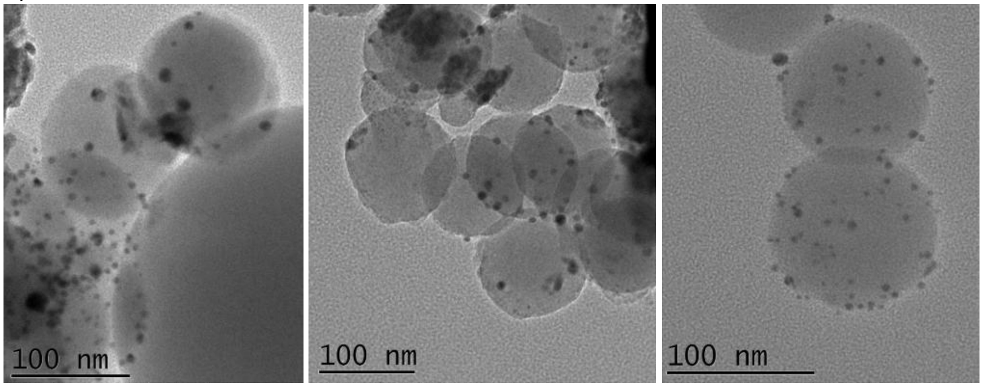

Figure S5. TEM micrographs of SiO_2-APTES-AuNPs nanostructures: a) Sample 1, b) Sample 2, and c) Sample 3.

6. Thermogravimetric analysis (TGA) of SiO_2 nanoparticles.

Thermogravimetric analysis of the SiO_2 nanoparticles was performed over the 28-500 °C range (Figure S6). Two mass-loss regions were observed. The first, between approximately 70 and 130 °C, corresponded to a mass loss of about 5.5% and is consistent with desorption of physically adsorbed water [1]. A second mass loss of approximately 6.1% occurred between 230 and 457 °C and may be associated with removal or decomposition of residual synthesis-derived species at the nanoparticle surface. Because the measurement ended at 500 °C, behavior above this temperature was not evaluated in the present TGA experiment.

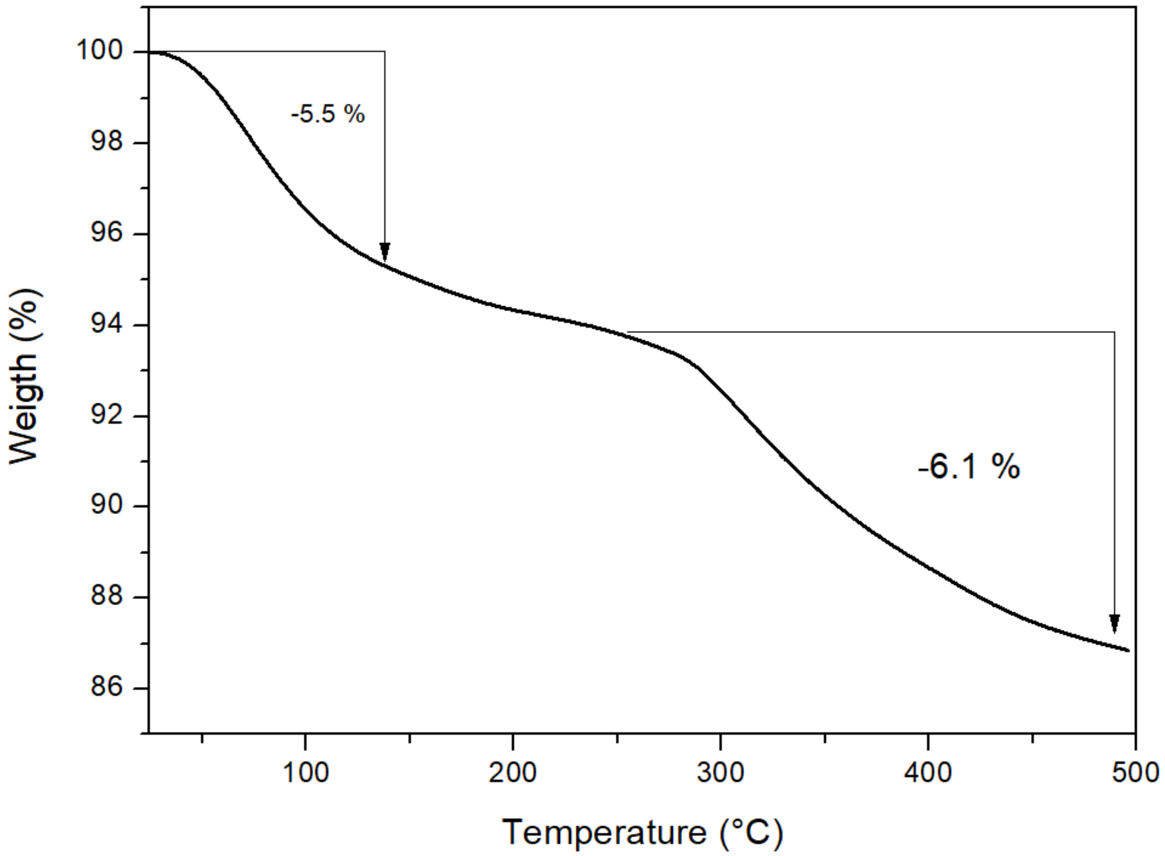

Figure S6. TGA of SiO_2 nanoparticles.

7. X-ray diffraction (XRD) of SiO_2 nanoparticles.

The XRD pattern of the SiO_2 nanoparticles is shown in Figure S7. A broad diffraction feature centered at 2θ = 23.41° is consistent with the predominantly amorphous structure of silica nanoparticles [1]. The broad profile, rather than sharp Bragg reflections characteristic of a crystalline phase, is consistent with the amorphous nature of SiO_2 [2,3].

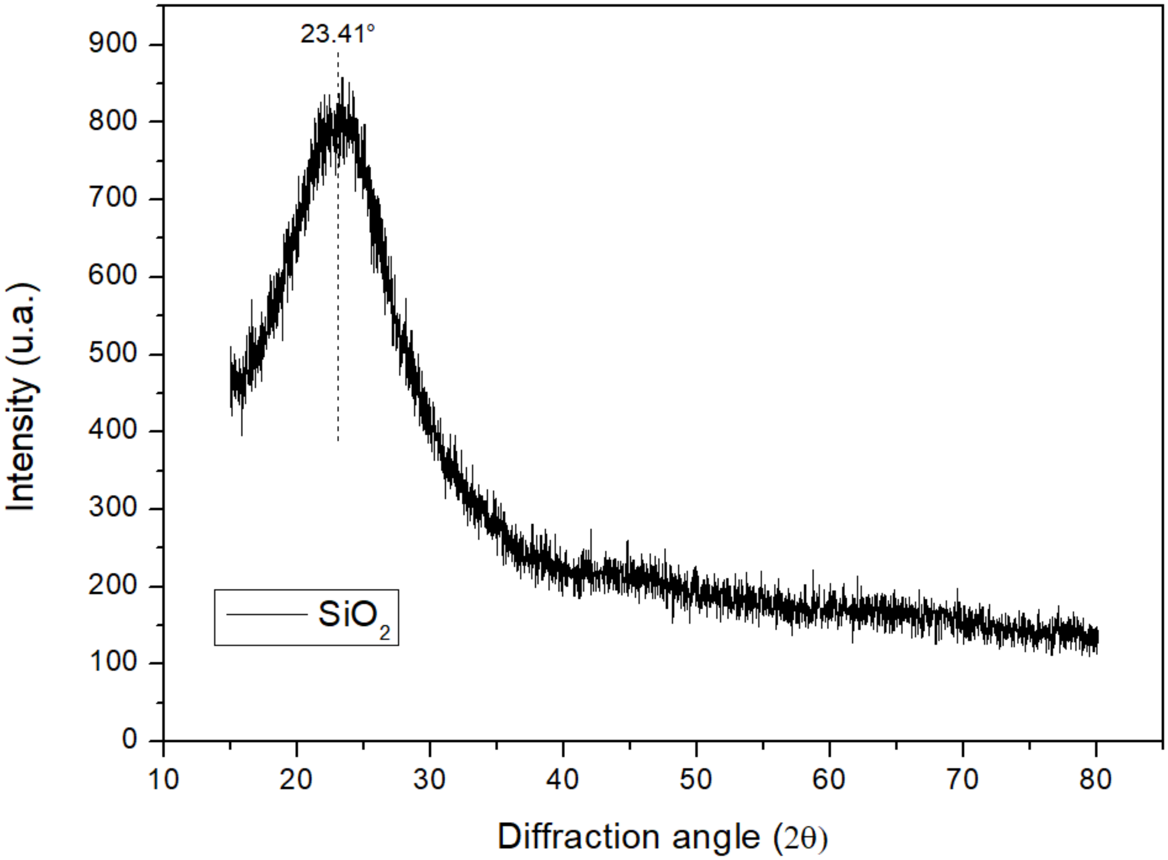

Figure S7. XRD pattern of SiO_2 nanoparticles.

8. DFT vibrational analysis

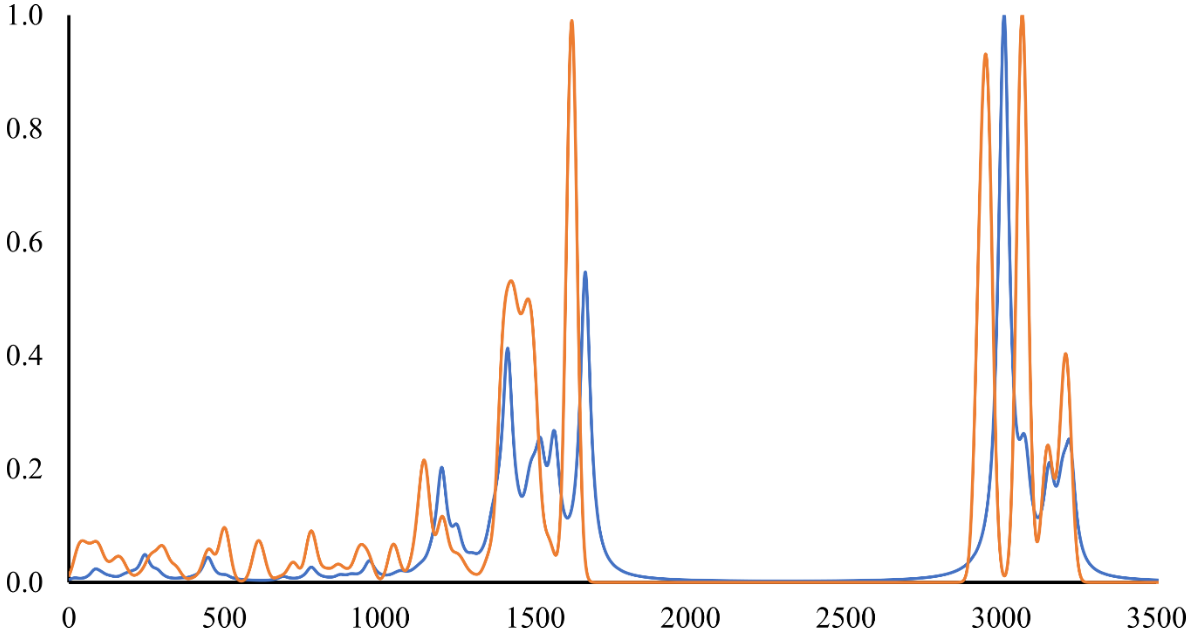

Figure S8. Raman spectra (constructed with DFT computed normalized activities) of MB (blue) and AuNP-MB (orange).

References

[1] Meky, N.; Salama, E.; Soliman, M.F.; Naeem, S.G.; Ossman, M.; Elsayed, M. Synthesis of Nano-silica Oxide for Heavy Metal Decontamination from Aqueous Solutions. *Water Air Soil Pollut.* **2024**, *235*, 154. https://doi.org/10.1007/s11270-024-06944-6.

[2] Biradar, A.I.; Sarvalkar, P.D.; Teli, S.B.; Pawar, C.A.; Patil, P.S.; Prasad, N.R. Photocatalytic degradation of dyes using one-step synthesized silica nanoparticles. *Mater. Today Proc.* **2021**, *43*, 2832–2838. https://doi.org/10.1016/j.matpr.2020.11.946

[3] Index of /guia_docente/backup/2014/469/45757/1. (s. f.-a). https://www5.uva.es/guia_docente/backup/2014/469/45757/1
